# Therapeutic Potential of Mesenchymal Stem Cell and Tenocyte Secretomes for Tendon Repair: Proteomic Profiling and Functional Characterization In Vitro and In Ovo

**DOI:** 10.3390/ijms26083622

**Published:** 2025-04-11

**Authors:** Petra Wolint, Iris Miescher, Asma Mechakra, Patrick Jäger, Julia Rieber, Maurizio Calcagni, Pietro Giovanoli, Viola Vogel, Jess G. Snedeker, Johanna Buschmann

**Affiliations:** 1 Division of Plastic Surgery and Hand Surgery, University Hospital Zurich, 8091 Zurich, Switzerland; petra.wolint@usz.ch (P.W.); iris.miescher@usz.ch (I.M.); julia.rieber@usz.ch (J.R.); maurizio.calcagni@usz.ch (M.C.); pietro.giovanoli@usz.ch (P.G.); 2 Institute for Biomechanics, ETH Zurich, 8092 Zurich, Switzerland; asma.mechakra@hest.ethz.ch (A.M.); patrick.jaeger@hest.ethz.ch (P.J.); jess.snedeker@hest.ethz.ch (J.G.S.); 3 Balgrist University Hospital, University of Zurich, 8008 Zurich, Switzerland; 4 Laboratory of Applied Mechanobiology, Department of Health Sciences and Technology, ETH Zurich, 8092 Zurich, Switzerland; viola.vogel@hest.ethz.ch

**Keywords:** mesenchymal stem cells, tenocytes, secretome, rabbit, tendon healing, angiogenesis, extracellular matrix, proteomics, CAM assay, angiogenic activity index

## Abstract

Tendon ruptures and tendinopathies represent a major part of musculoskeletal injuries. Due to the hypovascular and hypocellular nature of tendons, the natural healing capacity is slow and limited. Cell-free approaches for tendon injuries are being investigated as the next generation of therapeutic treatments. The aim of this study was to compare the proteomic profiles and biological activities of two different secretomes, obtained from New Zealand white rabbit adipose-tissue-derived mesenchymal stem cells (ADSCs) or a 3:1 mixed culture of ADSCs and rabbit tenocytes. The secretomes were analyzed by liquid chromatography–tandem mass spectrometry (LC–MS/MS) and their functional properties, such as gene expression, migration and angiogenesis, were investigated in vitro in rabbit tenocytes and in ovo using the chicken chorioallantoic membrane (CAM) assay after stimulation with secretomes or medium control. Both secretomes had a positive effect on angiogenesis and showed similar changes in relative gene expression levels associated with extracellular matrix (ECM) remodeling. Proteomic data showed that the two secretomes were clearly distinguishable, with 182 proteins significantly differentially expressed. The ADSC secretome was more effective in enhancing tenocyte migration under both healthy and inflammatory conditions. In the upregulated protein fraction of the mixed secretome, the tendon-related protein biglycan (BGN) and tenascin C (TNC) were increased. Based on our results, the mixed secretome shows great potential for promoting tendon healing as its composition is more effective in enhancing ECM-related processes and tendon development than the secretome of ADSCs.

## 1. Introduction

Tendons transmit muscle force to bone and bear high mechanical stress, often leading to injury and inflammation. Tendon rupture and tendinopathies account for a large proportion of musculoskeletal injuries, and four million new patients are diagnosed with tendon disorders each year worldwide, resulting in high economic costs [1,2,3]. Because tendons are hypovascular and hypocellular with low metabolic activity, their natural healing capacity is slow and inefficient [4]. Surgery, rehabilitation and pharmacological treatment are still the gold standard in clinics [5]. However, symptomatic treatment of pain and inflammation does not improve the recovery of tendons [6]. Tendon healing is a long process that takes months to a year and is often accompanied by fibrovascular scarring and adhesion formation. This leads to a deterioration in the mechanical and functional properties of the tendon [7] and often results in re-rupture in consequence [8]. Due to difficulties with treatment and the use of autografts and allografts, which are associated with problems such as the limited availability of suitable autologous tissue or immune-mediated rejection [2,9], research on synthetic or biological biomaterials has been intensified [10,11,12]. Polymers with integrated biomolecules [13], known as bioactive scaffolds, have shown promising results, such as a DegraPol tube as a delivery device for platelet-derived growth factor-BB (PDGF-BB) [14]. So far, however, none of these biomaterials has become established in the clinic [15]. In contrast, platelet-rich plasma (PRP) is a widely used autologous therapy in sports medicine [16] that is characterized by high levels of growth factors, cytokines and chemokines [17]. But there is a lack of standardization in the preparation and application of PRP [18].

Mesenchymal stem cells (MSCs) have been applied in several human clinical trials for tendon regeneration [19,20,21,22]. Efficacy was assessed by clinical parameters such as radiological examination and functional outcomes and showed overall improved tendon healing with a superiority of the cell-injected group compared to the control group [19,21]. The administration of MSCs nevertheless carries the risk of carcinogenesis [23,24,25], undesired ectopic tissue deposits [26], and impairment of tenogenic differentiation with concurrent administration of drugs such as dexamethasone [26,27]. In addition to MSCs, injected autologous expanded tenocytes also showed improvement in outcome in a case study [28]. However, the (long-term) cultivation of tenocytes is challenging, both in terms of obtaining sufficient numbers of cells and the fact that the cells undergo phenotypic loss and early senescence in vitro [29]. Co-culture with MSCs is a possible alternative as the influence of paracrine effects on the cells has been reported [30]. An altered gene expression of MSCs with higher scleraxis (*SCX*) and tenascin C (*TNC*) levels and an increased proliferation were observed [30].

A completely cell-free therapeutic approach is intended to increase safety for patients, while the efficacy of MSCs can be harnessed through secretomes for tendon healing. Factors released by the MSCs, such as growth factors, cytokines and vesicles, influence immunomodulatory and anti-apoptotic processes [31,32,33] and have already shown promising results in the treatment of injured tendons [31,34,35,36]. Inflammation acts as a negative regulator of tendon regeneration and is associated with a significantly higher rupture rate [37,38]. The immunomodulatory effect of human-bone-marrow-derived mesenchymal stem cell (BMSC) secretome has been demonstrated in an inflammatory environment in tendon–bone healing by inducing the M2 phenotype and inhibiting the M1 phenotype in macrophages by shifting in polarization status [37]. In addition, an increased fibroblast population appears to occur at the tendon–bone interface as a result of the modulated macrophage phenotype [37]. As a result, eight weeks after surgery, the rotator cuff enthesis in the rat model showed better biomechanical properties, including maximum failure load, stiffness and tension, eight weeks postoperatively than in the control group with medium alone [37]. Clinically, the application of secretome in spontaneous tendon and ligament injuries in a large animal model of horses led to a significant reduction in swelling in the cross-sectional area in the very early postoperative period and a lower re-injury rate [36]. Through trophic and paracrine mechanisms [28,39], the inhibition of lymphocyte proliferation was observed, leading to an anti-inflammatory effect and accompanied by improved functional and biomechanical outcomes in tendon and ligament healing using MSC-derived secretomes [40].

The beneficial effects of MSC-based therapies have not yet been fully elucidated, and the exact mechanism of action is still unclear [21]. To address this gap, several studies have characterized the secretome profile of MSCs [41,42,43], focusing on standardized production and quality control [44,45]. Proteomic analysis, such as two-dimensional liquid chromatography–tandem mass spectrometry (LC–MS/MS), provides information on the expressed proteins. Without a complete analysis of the secretome, the potential therapeutic agent remains a black box. To date, an effect on tendon regeneration has been demonstrated [31,34,35,36], but a defined composition of the secretomes produced and applied is lacking since MSC activity, and thus the properties of the MSC secretome, can be influenced by donor variability like age, sex, genetic predispositions and existing (chronic) diseases [46]. The difficulty in standardizing secretome production makes it more challenging to interpret the therapeutic effects and thus to identify key factors.

**Figure 1:**
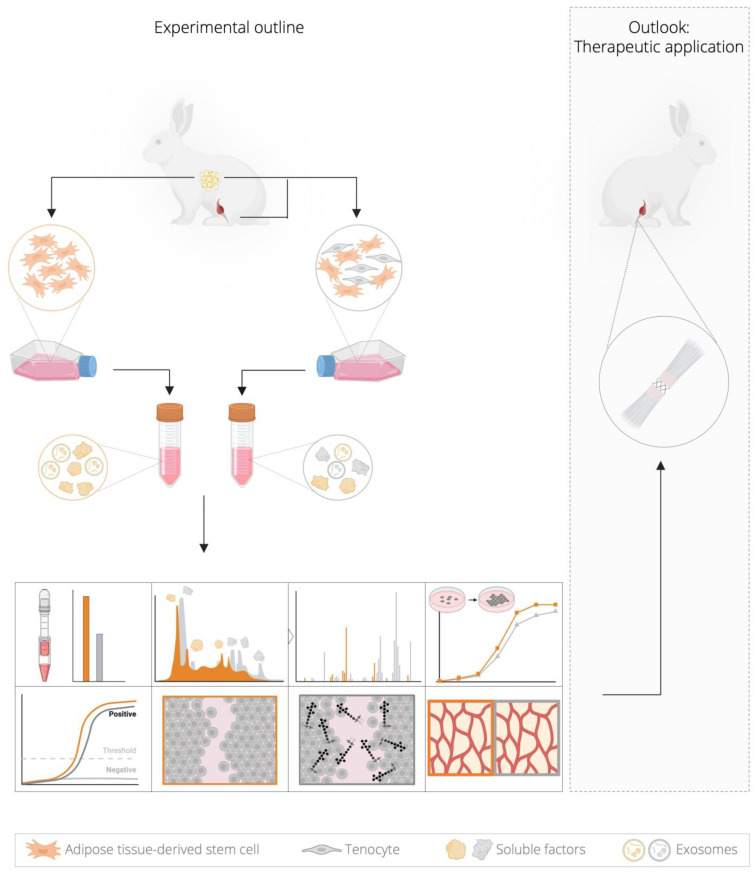
Experimental context for evaluating the therapeutic potential of the two secretomes using different assays. The schematic overview shows the tissue sources for fat and Achilles tendon in rabbits. The two cell types, rabbit-adipose-tissue-derived mesenchymal stem cells (rbADSCs) and rabbit tenocytes (rbTenocytes), were isolated from subcutaneous adipose tissue and Achilles tendon, respectively. The cells were maintained either in a pure rbADSC culture or in a mixed culture consisting of rbADSCs and rbTenocytes in a 3:1 ratio and served as the basis for the subsequent production of two different secretome types and the comparison for their protein composition and therapeutic potential. After two days of cultivation in serum-free medium, the supernatants were collected and the secretomes were concentrated using a dedicated filter. Both secretomes were analyzed in more detail through a comprehensive characterization by determining the total protein content and a proteomic profiling. Their mode of action on tenocytes was investigated using proliferation, gene expression, wound healing (including mimicking of a non-inflammatory and an inflammatory milieu) and angiogenesis assays. The results obtained provide insights into possible therapeutic applications for tendon healing after rupture. Graphic was created in https://BioRender.com.

The aim of the study (Figure 1) was to identify possible soluble proteins in two produced secretomes that could influence the ECM since the composition of the ECM has a non-negligible effect on the biomechanical properties and thus the stability of the regenerated tendons. This involves the comprehensive characterization of two secretomes to enable a systematic comparison in terms of (i) the characterization of the cells isolated from New Zealand white rabbit tissue, adipose-derived mesenchymal stem cells (ADSCs) and Achilles tenocytes, (ii) the proteomic profiling of the two concentrated secretomes from rabbit cells derived either from pure ADSCs or from a mixture of ADSCs and tenocytes in a 3:1 ratio, (iii) an evaluation of the efficacy in terms of the potential regenerative capacity of tendon cells in vitro by gene expression analysis, metabolic activity and migration assays after the stimulation of rabbit tenocytes with the respective secretome in a healthy and inflammatory environment and (iv) determining the angiogenic capacity in ovo by stimulation of chicken chorioallantoic membrane (CAM) for 7 days.

Combined proteomic profiling and functional analyses of the secretomes will provide the basis for a future treatment of the transected Achilles tendon with secretome in a rabbit model (Figure 1) to investigate the feasibility and efficacy of the cell-free therapeutic approach in vivo.

## 2. Results

### 2.1. Isolation of Primary Cells and Corresponding Characterization

**Figure 2:**
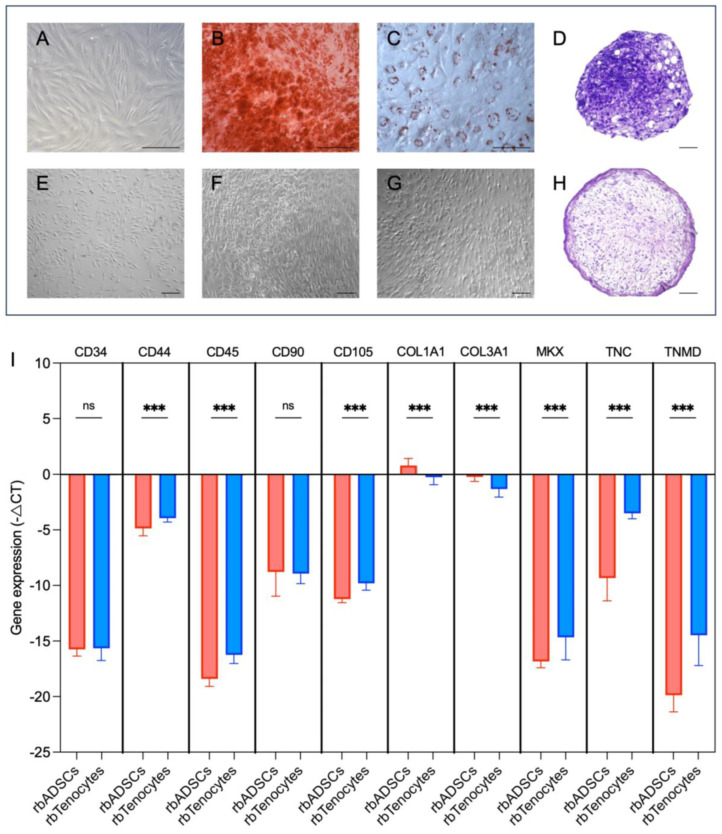
Differentiation and characterization of rabbit-adipose-tissue-derived mesenchymal stem cells and rabbit tenocytes. Rabbit-adipose-tissue-derived mesenchymal stem cells (rbADSCs; (**A**)) showed their differentiation capacities into the osteogenic (**B**), adipogenic (**C**) and chondrogenic (**D**) lineages. The unstimulated cells did not show any incipient differentiation in one of the investigated lineages due to cultivation in complete medium (**F**–**H**). Specific staining was used to detect differentiated cells using alizarin red staining for osteogenic differentiation (**B**,**F**), oil red-o staining for adipogenic differentiation (**C**,**G**) and toluidine blue staining for chondrogenic differentiation (**D**,**H**). Cell-type-specific markers for rbADSCs and rabbit tenocytes (rbTenocytes; (**E**)) were evaluated by quantitative real time polymerase chain reaction with the comparative deltaCT method (**I**). An upregulation of the expression of *CD90*, *CD105* and collagen 1A2 (*COL I*) was observed for rbADSCs compared to rbTenocytes, while lower levels were found for *CD34*, *CD44*, *CD45*, collagen 3A1 (*COL III*), homeobox protein mohawk (*MKX*), tenascin-C (*TNC*) and tenomodulin (*TNMD*). Conversely, tenocytes showed an upregulation of tendon-specific markers like *COL III*, *MKX*, *TNC* and *TNMD* (**I**). Data are expressed as mean ± SD. Groups were compared using nonparametric Kruskal–Wallis test (not significant (ns); *** *p* < 0.001). Scale bar 200 μm (**A**–**H**).

The ADSCs isolated from the adipose tissue of New Zealand white rabbits (Figure 2A) were examined for their differentiation potential after cultivation from passage 1. For all three donors, successful differentiation of the rabbit ADSCs (rbADSCs) into the adipogenic, osteogenic and chondrogenic lineages was confirmed by the corresponding staining with alizarin red, oil red-o and toluidine blue (Figure 2B–D,F–H), thus demonstrating the multipotent nature of the cells.

Both the rbADSCs and the isolated rabbit tenocytes (rbTenocytes) (Figure 2E) were analyzed for the classical markers of the two cell types. The gene expression profile of rbADSCs showed increased expression for *CD44*, *CD90*, *CD105*, collagen type I alpha 1 chain (*COL I*), collagen type III alpha 1 chain (*COL III*) and tenascin C (*TNC*) compared to the hematopoietic markers *CD34* and *CD45* and the tenogenic markers mohawk homeobox (*MKX*) and tenomodulin (*TNMD*; Figure 2I). The same markers were used to investigate the genotype of the rbTenocytes (Figure 2I). The rbTenocytes showed a significant upregulation (*p* < 0.001, nonparametric Kruskal–Wallis test) of the genes *CD44* (1.000 ± 0.060 vs. 1.960 ± 0.472 (rbADSCs vs. rbTenocytes, mean ± SD)), *CD45* (0.979 ± 0.251 vs. 4.680 ± 1.840), *MKX* (1.020 ± 0.198 vs. 3.840 ± 3.080), *TNC* (1.000 ± 0.027 vs. 51.2 ± 25.9) and *TNMD* (1.07 ± 0.44 vs. 176 ± 243) compared to the rbADSCs.

These results indicate an expected MSC phenotype and genotype for rbADSCs and confirm the expression of tendon-specific markers for the rbTenocytes (Figure S1).

### 2.2. Production of Two Types of Secretome and Comparative Analysis Using LC–MS/MS

Secretome production consisted of five cycles, which entailed the generation of secretome by the addition of serum-free medium and its harvest after 48 h, as well as a further feeding of the cells with complete medium for several days to restore the cells’ regular metabolism before the starvation phase was initiated again, followed by another secretome harvest. Cell viability was strictly monitored and measured after each cycle. It remained within a comparable range for both secretome types, which was 91.9 ± 5.8% for the rbADSCs and 89.3 ± 7.7% for the mixed culture of rbADSCs and rbTenocytes (ratio 3:1; rbMixed).

**Figure 3:**
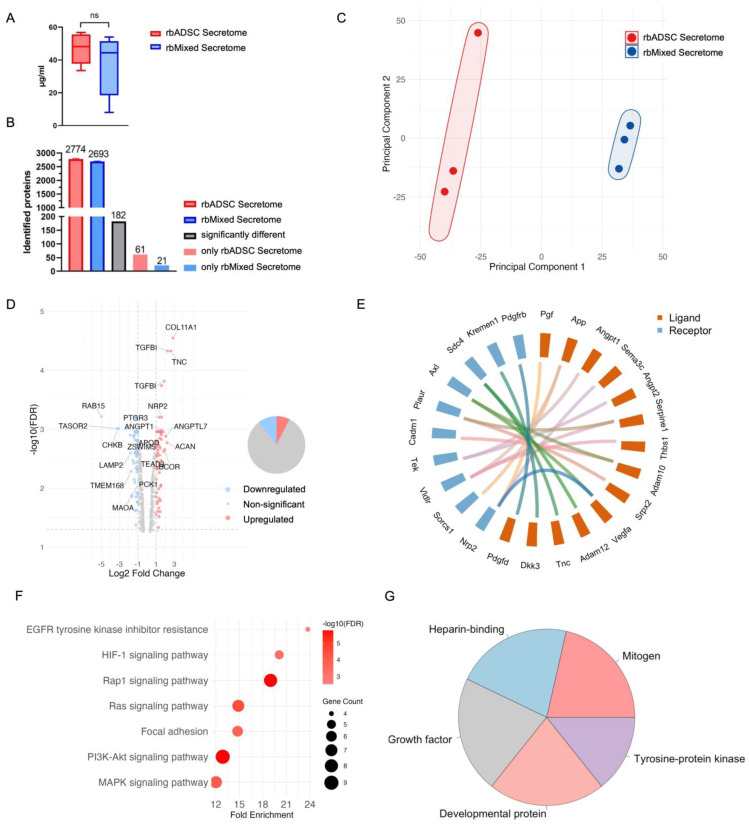
LC–MS/MS analysis of rabbit-adipose-tissue-derived mesenchymal stem cell (rbADSC) secretome and rbADSC and rabbit tenocyte mixed culture (ratio 3:1; rbMixed) secretome. Total protein concentration of rbADSC and rbMixed secretomes detected with BCA assay (**A**). Number of identified proteins (**B**). Principal component analysis (PCA) plot depicting the first and second principal components (PC1 and PC2; (**C**)). Normalized abundances were used as input. Volcano plot with −log10 transformed FDR as function of the log2 fold change (Log2FC) in protein expression between groups (**D**). The dashed gray vertical lines represent the Log2FC thresholds at ±1, indicating the cut-off for biologically significant expression changes. The horizontal threshold at FDR = 0.05 distinguishes significantly differentially expressed proteins, with colored dots highlighting those surpassing both criteria. Circus plot representing the paracrine network of ligand–receptor pairs of rbMixed secretome (**E**). Receptors are highlighted in orange and ligands in blue color. Abbreviations: Adam10 = a disintegrin and metalloproteinase domain containing protein 10, Adam12 = a disintegrin and metalloproteinase domain containing protein 12, Angpt1 = angiopoietin 1, Angpt2 = angiopoietin 2, App = amyloid precursor protein, Axl = AXL receptor tyrosine kinase, Cadm1 = cell adhesion molecule 1, Dkk3 = dickkopf 3, Nrp2 = neuropilin 2, Pdgfrb = platelet-derived growth factor receptor β, Pdgfd = platelet-derived growth factor D, Pgf = placental growth factor, Plaur = plasminogen activator, urokinase receptor, Sdc4 = syndecan 4, Sema3c = semaphorin 3c, Sorcs1 = sortilin-related VPS10 domain containing receptor 1, Srpx2 = sushi repeat containing protein X linked 2, Tek = TEK receptor tyrosine kinase, Thbs1 = thrombospondin 1, Tnc = tenascin-C, Vegfa = vascular endothelial growth factor A, Vldlr = very-low-density lipoprotein receptor. Ligand–receptor pairs enrichment analysis using DAVID KEGG pathways based on the reference genome oryctolagus caniculus (**F**). Pie chart of molecular function of identified ligand–receptor pairs of rbMixed secretome (**G**). Groups were compared using Shapiro–Wilk test (not significant (ns); (**A**)).

Total protein concentration was assessed prior to LC–MS/MS analysis and ranged from 20 to 50 µg/mL (Figure 3A). Around 2700 proteins could be identified in the secretomes, 182 of them being significantly differentially expressed. While 61 proteins were present only in the rbADSC secretome, 21 proteins were detected only in the rbMixed secretome (Figure 3B). Principal component analysis (PCA) demonstrated a clear distinction between the rbMixed and the rbADSC secretomes, with each secretome group analyzed in triplicate (Figure 3C). While the composition of rbMixed samples seemed to be quite homogeneous, one sample of the rbADSC secretome differed from the other two replicas. The protein abundance heat map confirmed the unequal protein expression of the two differently produced secretomes (Figure S2A). The volcano plot visualized that many proteins were above the defined threshold of FDR = 0.05 and had an expression difference smaller than −1 or larger than +1 being considered as relevant (Figure 3D and Figure S3A). In contrast to the rbADSC secretome, many of the upregulated proteins in the rbMixed secretome (Figure S2B) are present in the ECM, for instance, the proteoglycan aggrecan (ACAN) and BGN, the glycoprotein TNC, collagen XIA1 (COL XIA1) and MMP-9, playing a crucial role in ECM remodeling. Angiopoietin-1 (ANGPT1), on the other hand, plays an important role in vascular development and angiogenesis key processes during tendon healing. The most downregulated proteins compared to rbADSC secretome (Figure S2C) do not possess tendon-specific functions. Neither the ras-associated binding protein 15 (RAB15), choline kinase β (CKβ) nor fibronectin type III domain containing 3B (FNDC3B) were present in the rbMixed secretome, whereas amino oxidase (AMO) was identified in both secretomes but less expressed in the rbMixed secretome group. Although the secretomes were clearly distinctive, most of the proteins were equally expressed (Figure S2D), amongst them proteins concerning ECM as COL I, COL III, TIMP metallopeptidase inhibitor 1 (TIMP1), growth factors relevant for tendon healing, cytokine transforming growth factor beta 1 (TGF-β) and pro-inflammatory marker interleukin-6 (IL-6).

To discover paracrine interactions in rbMixed and rbADSC secretome, we programmatically extracted ligand–receptor pairs from our secretome data using a curated database as a reference [47]. While several pairs were differentially upregulated in the rbMixed secretome, no pairs were differentially downregulated (Figure 3E and Figure S3B). KEGG pathway analysis using DAVID database [48] revealed the enrichment signaling pathways like Ras, Rap1, PI3K Akt, MAPK and HIF-1 in the rbMixed secretome based on the ligand–receptor analysis. These pathways influence proliferation, differentiation, cell growth, apoptosis and survival, inflammation, metabolism and angiogenesis amongst other functions (Figure 3F). The molecular function of these interactions was assigned to growth factors, mitogens, developmental proteins, heparin-binding and tyrosine-protein kinases (Figure 3G).

### 2.3. Over-Representation Analysis (ORA) of Secretome Proteins

**Figure 4:**
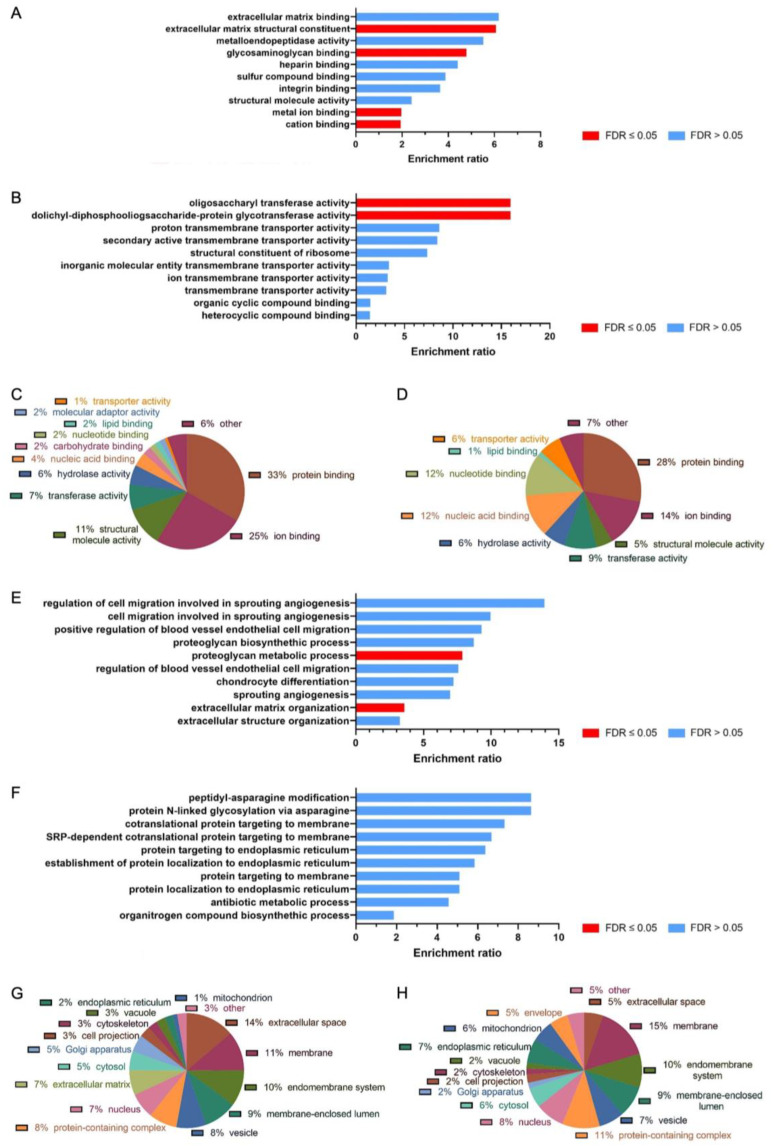
Over-representation analysis (ORA) of rabbit-adipose-tissue-derived mesenchymal stem cell (rbADSC) and rabbit tenocyte mixed culture (ratio 3:1; rbMixed) secretome compared to rbADSC secretome. Enrichment ratio of gene ontology molecular function of upregulated proteins (**A**) and of downregulated proteins (**B**) in rbMixed secretome compared to rbADSC secretome. Activities at molecular level of upregulated proteins (**C**) and of downregulated proteins (**D**) in rbMixed secretome compared to rbADSC secretome. Enrichment ratio of gene ontology biological process accomplished by multiple molecular activities of upregulated proteins (**E**) and of downregulated proteins (**F**) in rbMixed secretome compared to rbADSC secretome. Pie chart of location, relative to cellular compartments and structures of upregulated proteins (**G**) and of downregulated proteins (**H**) in rbMixed secretome compared to rbADSC secretome. Parameters for the enrichment analysis: minimum number of IDs in the category: 5; maximum number of IDs in the category: 2000; FDR method: BH; significance level: Top 10.

In order to identify enriched molecular functions and biological processes, lists of upregulated and downregulated proteins in the rbMixed secretome compared to rbADSC secretome were analyzed using the WEB-based GEne SeT AnaLysis Toolkit [49]. Molecular functions of upregulated proteins could be associated with ECM functions (Figure 4A). Significant enrichment ratios were detected for ECM structural constituent, glycosaminoglycan (GAG) binding and metal and cation ion binding. Molecular functions of downregulated proteins (Figure 4B) showed enrichment ratios mainly in transferase activity and transmembrane transporter activity but not in tendon-healing-related functions. Comparing the different compartments of molecular functions in upregulated proteins (Figure 4C) and downregulated proteins (Figure 4D), the proportions of some compartments varied. While clearly more ion binding and, to some extent, also protein binding and structural molecule activity in the upregulated proteins were detected, nucleic acid and nucleotide binding were much more prominent in the downregulated protein fraction.

The enrichment of biological processes in upregulated proteins in rbMixed secretome concerned also ECM as a proteoglycan biosynthetic process, and ECM organization had significantly higher enrichment ratios (Figure 4E). Higher but not significant enrichment ratios were also detected for angiogenesis, endothelial cell migration in connection with blood vessels and chondrocyte differentiation. In contrast to the upregulated proteins, no significant enrichment ratio was detected in the downregulated proteins (Figure 4F). Categories of biological processes are visualized in Figure S4. Cellular component analysis showed that extracellular matrix proteins were present only in the upregulated protein fraction, and more gene products were in the extracellular space than in the membrane (Figure 4G), while the opposite was the case for the downregulated proteins (Figure 4H). Looking at the cell organelles, more gene products in the upregulated protein fraction were localized in the Golgi apparatus than in the endoplasmic reticulum and mitochondria, whereas the trend was reversed in the downregulated protein fraction.

### 2.4. Gene Expression of Tenocytes After Stimulation with Corresponding Secretomes

**Figure 5:**
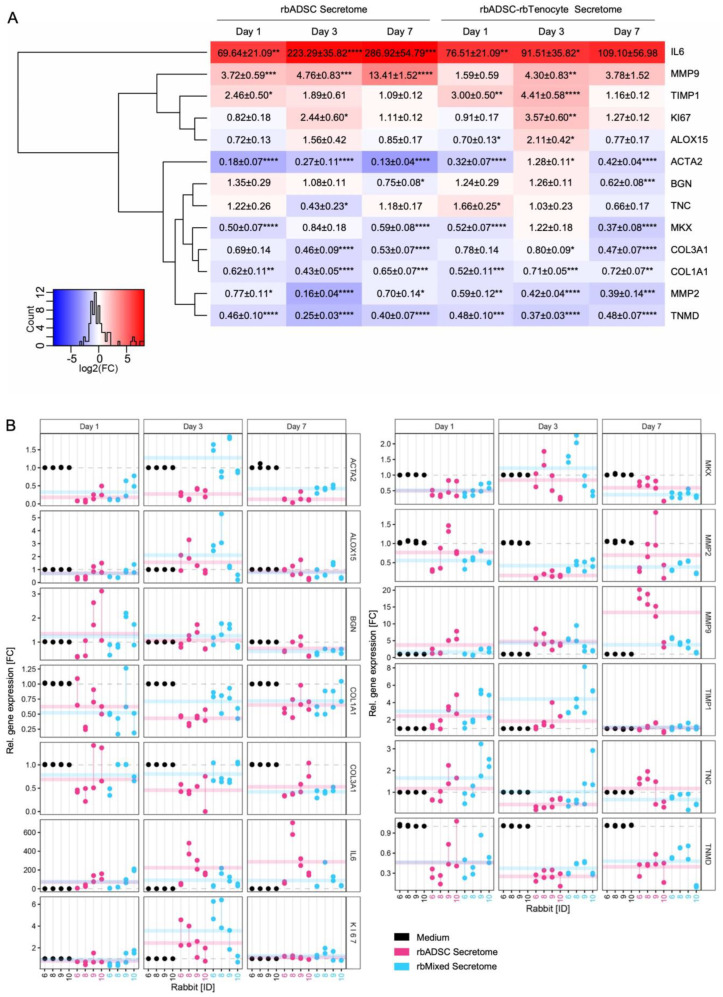
Gene expression of extracellular matrix, tendon-specific and inflammatory-related markers and of proliferation marker *Ki67*. Rabbit tenocytes from 4 donors (Nos. 6, 8, 9 and 10) were cultured in rabbit-adipose-tissue-derived mesenchymal stem cell (rbADSC) secretome, rbADSC and rabbit tenocyte mixed culture (ratio 3:1; rbMixed) secretome or in medium (control) for 1 day, 3 or 7 days. Experiment was performed twice. To analyze qPCR data, a linear mixed model was applied with fold change as a response variable. Medium type was set as fixed and rabbit as random effect for genes. Heat map of upregulated or downregulated genes in rbMixed secretome and rbADSC secretome compared to medium (**A**). Values are shown as mean fold change (FC) ± standard error of the mean (SEM). P-value adjustments were carried out with Benjamini–Hochberg correction (significance: * *p* ≤ 0.5, ** *p* ≤ 0.01, *** *p* ≤ 0.001 and **** *p* ≤ 0.0001). Overview of relative gene expression at days 1, 3 and 7 as fold change compared to medium, set to 1 (**B**).

Several genes were analyzed concerning gene expression alteration caused by the exposition to rbADSC or rbMixed secretome compared to cells in medium (control). A heat map shows the statistical analysis and clustering according to similar fold changes of gene expression (Figure 5A), whereas the results of single experiments are visualized as dots in Figure 5B. The pro-inflammatory marker *IL-6* was strongly upregulated in both secretomes compared to medium at every time point. However, in rbMixed-secretome-treated tenocyte levels were significantly lower at days 3 and 7 than in rbADSC secretome cultures. At day 7, *IL-6* expression level was 2.6-times lower with the rbMixed than with the rbADSC secretome and was not significantly different from control levels in medium. In contrast, the inflammatory resolving marker *ALOX15* showed a slight downregulation on days 1 and 7 for both secretomes. On day 3, a weak but significant upregulation for rbMixed secretome treated tenocytes could be observed, which was 1.4-times higher than in rbADSC secretome. Besides *IL-6*, most pronounced upregulation has been found for *MMP-9*, with the rbADSC secretome showing increasing expression levels over time that were 3.6-times higher than those observed in tenocytes incubated with rbMixed secretome on day 7. Levels for *MMP-9* with rbMixed secretome treatment peaked at day 3 with a significantly higher result than in control cells, whereas on days 1 and 7, the results did not differ significantly from control condition. The *MMP-9* inhibitor *Timp1* showed an upregulation on day 1 and on day 3 being most pronounced at day 3 for rbMixed secretome with a 2.3-times higher value than tenocytes cultured in rbADSC secretome. On day 7, expression levels with both secretomes were nearly equal to tenocytes cultured in medium. In contrast to *MMP-9*, *MMP-2* was downregulated at every time point for both secretomes with the strongest downregulation for rbADSC secretome at day 3. In addition to *MMP-2*, many other genes regarding ECM and typical tendon markers were reduced. Strong downregulation was observed for *α-SMA* (*ACTA2*), a marker for myofibroblasts. Reduction was more pronounced for rbADSC secretome with a 3.3-times lower fold change compared to rbMixed secretome at day 7. Like α-SMA, *COL I* and *COL III* were also downregulated, but treatment effects were more similar. Additionally, typical tendon markers like *MKX* and *TNMD* showed significantly reduced expression levels for each measurement, in contrast to *TNC* for which fold changes were mostly like values in control cells. Similarly, expression levels of the proteoglycan *BGN* were often equal to control conditions, although a significant fold change reduction could be observed on day 7. The proliferation marker *Ki67* was moderately but not significantly reduced on day 1, revealed a significant upregulation on day 3 and showed similar expression levels like control tenocytes for both secretome groups at day 7. Like for *Ki67*, expression levels of α-SMA (*ACTA2*), *ALOX15* and *MKX* at day 3 differed compared to day 1 and to day 7. Also, fold change differences between rbADSC and rbMixed-secretome-treated tenocytes were often detected on day 3. Some markers like α-SMA, *TNC* and *MKX* were downregulated in rbADSC secretome but upregulated in rbMixed secretome, though results were not always significantly different from controls. Other markers such as *TIMP1*, *Ki67*, *ALOX15* and *BGN* revealed an upregulation in both conditions, but effects were stronger for rbMixed secretome. In contrast, *TNMD*, *COL I*, *COL III* and *MMP-2* were downregulated in both conditions, but downregulation was weaker in the rbMixed secretome group.

### 2.5. Improved Wound Healing In Vitro

**Figure 6:**
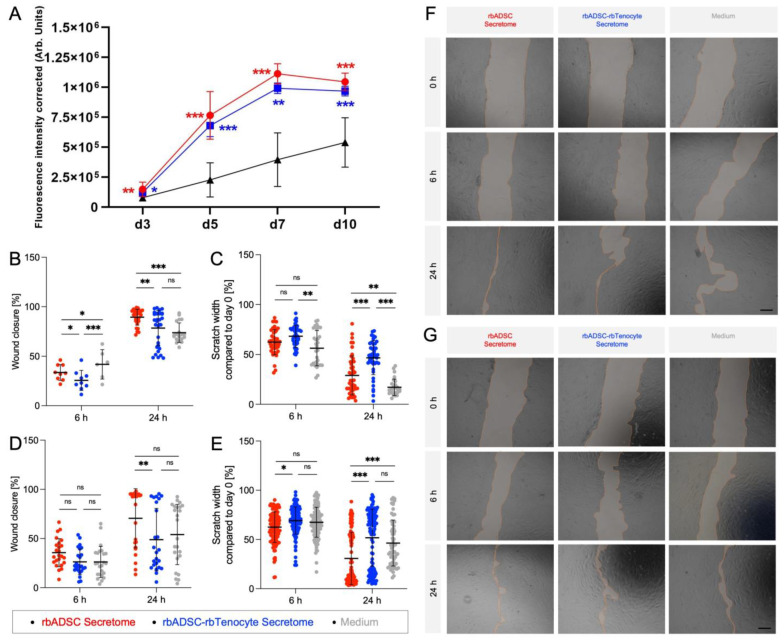
The effect of secretomes on metabolic activity and rabbit tenocyte migration in vitro. Cell viability assay using rabbit tenocytes cultured in secretome or medium (**A**). In the in vitro wound-healing assay, healthy rabbit tenocytes were treated with either secretome of rabbit adipose tissue-derived mesenchymal stem cell (rbADSC) culture, secretome of rbADSCs with rabbit tenocytes (in a ratio 3:1) culture or complete medium as a control for 6 h and 24 h (**B**,**C**,**F**). The same treatment with secretomes was performed under inflammatory conditions by stimulating the cells with lipopolysaccharide (LPS; (**D**,**E**,**G**)). Representative phase contrast images are shown in (**F**,**G**) (scale bar 200 μm). The area of percent wound closure was assessed with a microscope and calculated in comparison to the scratch on day 0 (**B**,**D**). In addition, the change in scratch width over 6 h and 24 h was analyzed (**C**,**E**). Groups were compared using nonparametric Kruskal–Wallis test (**A**) and using two-way ANOVA (mixed model) with Geisser–Greenhouse correction and Tukey’s post hoc correction for multiple comparison ((**B**–**E**); (not significant (ns); * *p* < 0.05; ** *p* < 0.01; *** *p* < 0.001). Values shown as mean fold change ± SD (**B**–**E**). The control group is shown in gray, the rbADSC secretome group is shown in red and the rbADSC–rbTenocyte secretome group is shown in blue (**A**–**E**).

A significantly higher viability of rbTenocytes cultured with either of the two secretomes, produced from rbADSC culture or from rbADSCs and rbTenocytes in a 3:1 ratio culture, compared to the control with rbTenocytes in medium. No difference was detected between the two secretome groups. A plateau was reached on day 7 for the tenocytes cultured with secretomes (Figure 6A).

The wound-healing capacity of the two secretome groups was tested on rbTenocytes using the scratch assay. The cells were either exposed to medium, which served as a control, or to one of the two secretomes, rbADSCs only and rbADSCs and rbTenocytes in a ratio of 3:1 (Figure 6B–G). The cells treated with rbADSC secretome showed a significantly greater wound closure after 24 h compared to the cells cultivated with the mixed secretome (*p* < 0.01) and with the medium (*p* < 0.001; Figure 6B). The mixed secretome showed a wound closure of 78.4 ± 17.8% (mean ± standard deviation (SD)) and was not able to convince with significance compared to the control (73.7 ± 9.87%). At the earlier time point, 6 h, the wound-closure values of the three groups were between 25.6% and 41.8% (Figure 6B). In contrast to the change in total wound area, the control group, which was only treated with medium, showed a significant reduction in scratch width compared to the samples treated with one of the two secretomes (Figure 6C). After 24 h, the control group had a scratch width of 17.1 ± 8.24% compared to the baseline measurement, the rbADSC secretome group 29.0 ± 19.6% and the rbADSC and rbTenocyte secretome group 46.7 ± 16.9% (Figure 6C).

To mimic the effect on wound healing in an inflammatory environment in vitro, the rbTenocytes were exposed to lipopolysaccharide (LPS; Figure 6D,E,G). After treatment with either medium as a control or with one of the two secretomes, wound closure was determined again. This showed a slightly different trend than without LPS treatment with no significant differences after 6 h and increased wound closure after 24 h in rbTenocytes treated with rbADSC secretome compared to cultures treated with mixed secretome (*p* < 0.01) and medium. In terms of scratch width, the cells treated with rbADSC secretome were convincing at an earlier time point with a decreasing value compared to the other two groups. After 24 h, this group still showed the lowest value for the width (30.8 ± 26.6%) of all three groups (mixed secretome: 51.8 ± 29.0%, medium control: 41.1 ± 25.0%; Figure 6E).

Compared to the cultures treated with LPS, the values for wound closure in the rbTenocyte cultures without LPS showed an overall more homogeneous distribution with a higher wound closure capacity. A less favorable wound closure was observed across the board in the samples treated with LPS. This trend was not as pronounced for the scratch width parameter. The cultures treated with rbADSC secretome showed an increased wound-healing ability for both parameters and both conditions, without and with LPS, compared to the other two groups, the control and the rbADSC and rbTenocyte mixed secretome.

### 2.6. Increased Angiogenic Potential In Ovo

**Figure 7:**
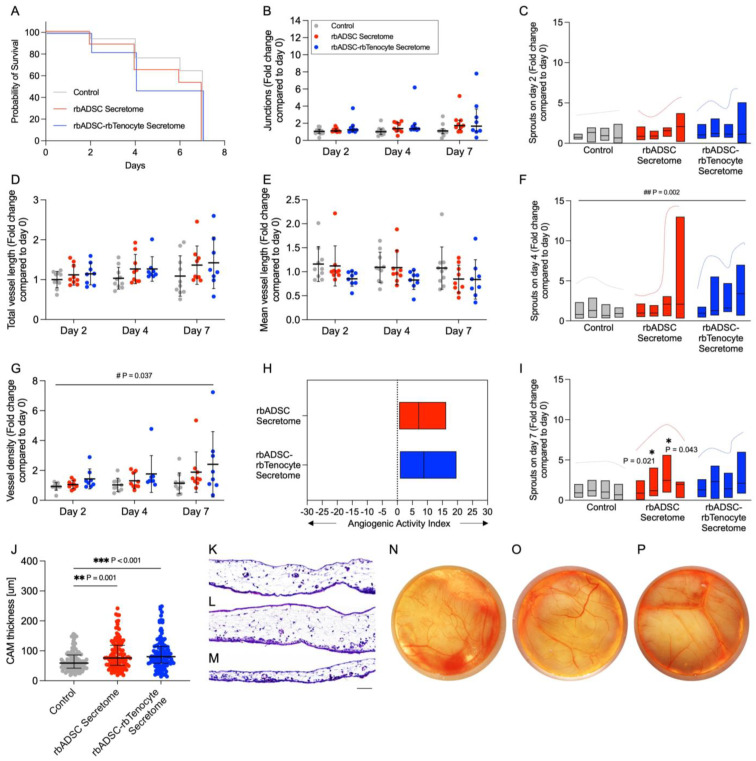
Comparison of the two secretomes for angiogenic capacity. The chicken chorioallantoic membrane (CAM) was treated for 7 days with either a secretome from rabbit-adipose-tissue-derived mesenchymal stem cell (rbADSC) culture, a secretome from rbADSCs with rabbit tenocyte (in a 3:1 ratio) culture or serum-free medium as a control (**A**). The junctions (**B**), vessel hierarchy (**C**,**F**,**I**), total vessel length (**D**), mean vessel length (**E**) and vessel density (**G**) were determined on days 2, 4 and 7 of treatment and the values show the fold change compared to day 0 before treatment. Vessel hierarchy includes primary, secondary, tertiary and quaternary sprouts, shown as bars in this order from left to right for each group (**C**,**F**,**I**). The dashed line symbolizes the trend in the extent of branching. On day 7, the CAM was removed, and the thickness (**J**) was measured using H&E-stained sections (rbADSC secretome (**K**), rbADSC–rbTenocyte secretome (**L**) and control (**M**); scale bar 50 μm). Based on the parameters’ probability of survival (**A**), junctions (**B**), vessel hierarchy (**I**), total vessel length (**D**), vessel density (**G**) and CAM thickness (**J**), the angiogenic activity index (AAI) was calculated (**H**), which includes the control group as a reference (value 0), and an index with a value of 1 indicates a doubling of angiogenic activity, whereas an index of −1 indicates a decrease in angiogenesis to half compared to the control group. Exemplary microscopic image of the vascularized and treated CAM with rbADSC secretome (**N**), rbADSC–rbTenocyte secretome (**O**) and serum-free medium (**P**). Values shown as mean fold change ± SD (**B**,**D**,**E**,**G**), median fold change ± interquartile ranges (**J**) or min to max bars with median (**C**,**F**,**I**). The control group is shown in gray, the rbADSC secretome group is shown in red and the rbADSC–rbTenocyte secretome group is shown in blue (**A**–**J**). Groups were compared using log-rank (Mantel–Cox) test (**A**), two-way ANOVA (mixed model) with Geisser–Greenhouse correction and Tukey’s post hoc correction for multiple comparison (hash indicates p values for comparison of stimulation across time points (**G**) and across sprouts (**F**)), Mann–Whitney test (* *p* < 0.05; comparison of day 7 to day 2; (**I**)) and nonparametric Kruskal–Wallis test (** *p* < 0.01; *** *p* < 0.001; (**J**)). Values are not significant unless otherwise stated.

Comparable survival was observed in chicken embryos between groups (Figure 7A). Microscopic images of the chorioallantoic membrane (CAM) of the treated area with one of the two secretomes or the control group were used to determine the number of junctions (Figure 7B), total vessel length (Figure 7D) and vessel density (Figure 7G). An increase in the values of these parameters over time was observed in all groups. To consider the initial vascularization, the values were calculated as a fold change compared to the vascularization of the CAM before treatment. On day 7, a 1.19 ± 0.72 fold change (mean ± SD) was observed for the number of junctions in the control group, for the rbADSC secretome group 2.07 ± 1.26 and for the rbADSC–rbTenocyte secretome group 2.54 ± 2.42. For the total vessel length, a fold change of 1.09 ± 0.51 for control group, 1.36 ± 0.48 for the rbADSC secretome group and 1.42 ± 0.65 for the rbADSC–rbTenocyte secretome group was found for the three groups. Vessel density values on day 7 showed 1.15 ± 0.70 fold change for control, 1.45 ± 0.41 fold change for rbADSC secretome and 2.41 ± 2.19 fold change for rbADSC–rbTenocyte secretome. The mean vessel length showed a decrease over time for all three groups (Figure 7E). Vessel hierarchy was assessed by the degree of sprouting. The number of sprouts for the first to fourth degree of branching was determined. While the control group showed an equal degree of branching for the second to fourth degree of branching on day 2 of treatment, an indication of a more pronounced number of second-degree branches was observed on days 4 and 7. In the rbADSC–rbTenocyte secretome group, the same trend was observed throughout the treatment period, indicating an increase in second-degree branching and an increase in small fourth-degree blood vessels. In contrast, the vascular hierarchy of CAMs treated with the rbADSC secretome showed a changing trend over time. While fourth degree blood vessels dominate on days 2 and 4, the expression shifts to second- and third-degree branches on day 7. After treatment, CAM thickness was measured (Figure 7J–M), which revealed a significant increase in CAM size in the secretome treatment groups compared to the control group (59.0 μm (median) for the control group, 76.2 μm for the rbADSC secretome group and 80.5 μm for the rbADSC–rbTenocyte secretome group).

The angiogenic activity index (AAI) [50] was calculated based on the values for junctions, total vessel length, vessel density and hierarchy. The control group was used as a reference for natural vascularization and the angiogenic capacity of the two secretome groups was compared. Both groups showed pronounced angiogenic activity, with the rbADSC secretome showing an average of 715% (index 7.15) increased angiogenesis compared to the control group. The rbADSC–rbTenocyte secretome was convincing with 885% increased angiogenesis (index 8.85; Figure 7H).

## 3. Discussion

**Figure 8:**
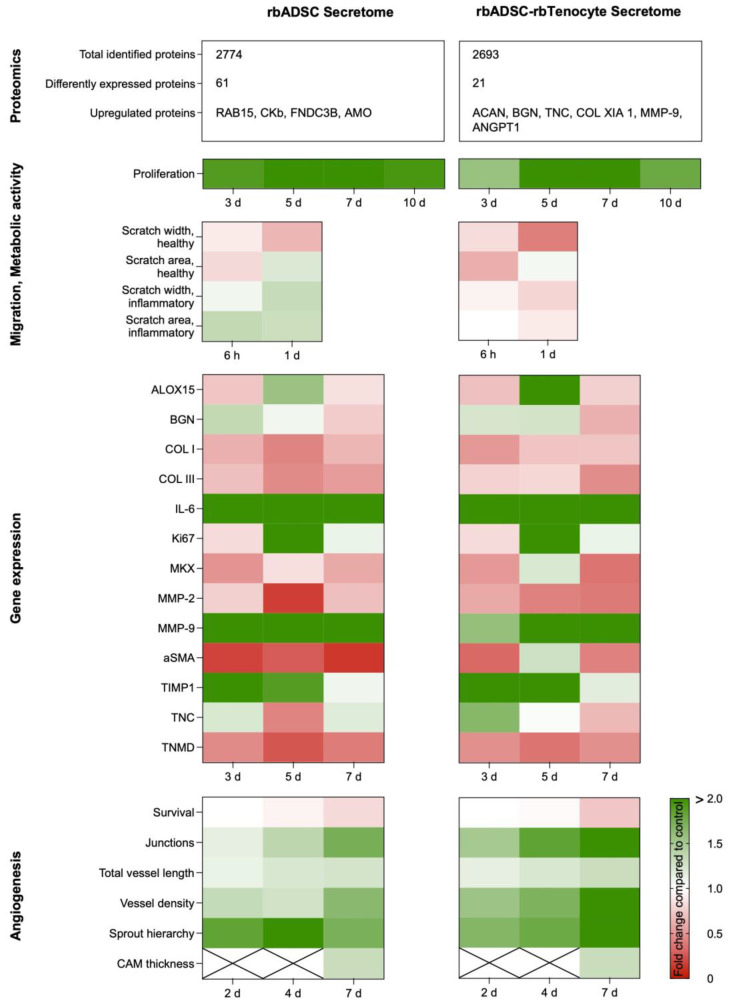
Summary of the comparison of the two secretome types based on rabbit-adipose-tissue-derived mesenchymal stem cells (rbADSCs) and rbADSCs with rabbit tenocytes co-culture (rbADSC–rbTenocyte). Heat maps of the results of the functional activities. Values represent the fold change of the mean value compared to the control group. A value of 1 indicates no change compared to the control group.

Cell-free approaches for tendon injuries are being investigated as the next generation of therapeutic treatment options. In this study, two different secretomes, from an rbADSC culture and from a mixed culture based on rbADSCs and rbTenocytes in a 3:1 ratio, were examined and compared regarding their proteomic profiles and biological activities (Figure 8, Table S1). This included the influence of the two secretomes on the ECM remodeling, proliferation and migration capacity of tenocytes, as well as their gene expression after treatment with the secretomes. The impact on neovascularization was evaluated by using the CAM assay; and, based on the results, an angiogenic profile was established for both secretome types.

The result of our study demonstrates that the secretome from combined culture of rbADSCs with rbTenocytes affects the proteomic profile differently in part compared to secretome produced by rbADSCs alone (Figure 3B–D). We observed that rbMixed secretome contained more ECM-relevant proteins important for tendon functionality (Figure S2B) as the proteoglycans aggrecan and biglycan [51] and the glycoprotein tenascin-C [52] were upregulated (Figure S2B). This result reflects the presence of tenocytes in the rbMixed culture as these fibroblast-like cells produce ECM, such as collagen, fibronectin and proteoglycans [53]. Furthermore, the paracrine network analysis identified the tenascin-C receptor in the rbMixed secretome H. While there was a differential overexpression of tenascin C protein in the rbMixed secretome compared to the rbADSC secretome, the supplementation of the two secretomes to a tenocyte culture induced a further induction of TNC gene expression on day 3 when the rbMixed secretome was used, with a decline to a downregulation on day 14—which was not observed when the rbADSC secretome was applied (there was only a slight increase in TNC gene expression on day 3 with a pronounced downregulation afterwards).

Tenascin C is an ECM component with multiple functions [54]. Generally speaking, tenascin C is reported to bind to fibronectin, proteoglycans and collagen in the ECM of tendons and furthermore act as a reservoir for many growth factors and other soluble factors because they bind to tenascin C in the ECM. Moreover, tenascin C protein can bind to cell surface receptors for a “direct” communication with the cells; by EGFR (activating proliferation), TLR4 (activating inflammation), syndecan 4 (blocking fibronectin-mediated cell adhesion, resulting in an anti-adhesive effect), among others; and it can bind to the cell surface also by various integrin binding sites, thereby activating cell signaling primarily affecting mechanical interactions via cell motility or contractions as well as adhesion modulation.

Thus, an abundance of tenascin C protein applied to an acute tendon injury, such as a ruptured tendon, via secretome injection, may not only offer an initial in situ structural support by binding with other ECM components like fibronectin or collagen but also restrict the adhesion formation of the healing tendon to the surrounding tissue, which may be particularly important in flexor tendon repair with an injured synovial sheath as flexors are especially prone for adhesion formation [55]. Because we found that the rbMixed secretome induced further TNC gene expression in tenocytes (at least at day 3 in vitro), it can be expected that the application of such secretome in vivo would also lead to an increase in tenascin C protein levels, leading to the functional impacts discussed above.

The growth factor PDGF found in this analysis H has been shown to positively influence tendon healing as it affects matrix remodeling and increases collagen synthesis and cell proliferation [56].

Tendons are exposed to high mechanical stress and continuous adaptation to strain is crucial; therefore, ECM remodeling is an important process during tendon homeostasis and healing, regulated by zinc-dependent metalloproteases (MMPs) and their inhibitors Timp [53,57,58]. In contrast to the similarly expressed MMP-14 and Timp1 (Figure S2D), more MMP-9 protein was detected in the rbMixed secretome (Figure S2B).

MMP-9 belongs to the family of metalloproteases that are able to degrade the ECM components. To keep MMP activity in check, their inhibitors (TIMPs) are expressed by the cells, which do not only inhibit MMPs but also restrict TGFβ1 expression and thus may reduce fibrosis [59]. The balance between MMPs and TIMPs is crucial for homeostasis as well as for wound healing [60].

In the rbMixed secretome, there was a differential overexpression of MMP-9 compared to the rbADSC secretome. Interestingly, both secretomes applied to a tenocyte culture induced a prominent MMP-9 gene upregulation. An application of these secretomes to a ruptured tendon would, therefore, directly provide MMP-9 protein (rbMixed) or induce MMP-9 gene expression (both secretomes)—and impact particularly the remodeling phase during tendon healing. As ruptured Achilles tendons have been reported to exhibit a significantly higher MMP-2 and MMP-9 gene expression at the rupture site than healthy tendons [57], our secretomes would further accentuate this natural increase as a response to injury. Degradation of debris structural components may in turn be accelerated, the remodeling phase shorter and, as a consequence, the consolidation phase with the collagen alignment earlier. As we found a simultaneous TIMP1 gene upregulation under both secretome supplementation to tenocytes, a further interpretation is that cells react to the MMP-9 overexpression, particularly prominent in the rbMixed secretome, where a TIMP1 gene upregulation in the tenocyte in vitro culture was still active on day 7, whereas it declined after day 5 already in the rbADSC-secretome-treated tenocytes.

Overall, the provision of MMP-9 via rbMixed secretome to an acute tendon injury is potentially connected to a faster remodeling phase and returned to a regenerated state earlier.

Higher MMP-9 protein expression in the rbMixed secretome is furthermore consistent with the over-representation analysis of molecular functions in which significant enrichment ratios were observed in the rbMixed secretome for “extracellular matrix structural constituent”, “metal ion binding” and “cation binding” (Figure 4A). Additionally, activities at the molecular level (Figure 4C,D) showed that there was more “ion binding” in the upregulated protein fraction compared to the downregulated protein fraction (25% vs. 14%) and that higher values for “structural molecule activity” were obtained (11% vs. 5%). Secretome treated tenocytes showed increased relative gene expression of *MMP-9* for both secretomes (Figure 5A), but the effect was significantly stronger for the rbADSC secretome having an approximately 13.4-times higher result compared to the control cells and to cells treated with rbMixed secretome (3.8-times higher values). In contrast, significantly lower gene expression levels were measured for *MMP-2* for both secretomes at every time point with a strongest reduction for rbADSC secretome at day 3 (6-times lower). The tissue inhibitor of metalloproteases *Timp1* revealed a slight increase with highest value at day 1 for the rbADSC secretome (2.5-times higher) and at day 3 for rbMixed secretome at day 3 (4.4-times higher). Therefore, the increased relative gene expression of *MMP-9* might be accompanied by a weakening of the collagen structure of the tendon influencing mechanical properties negatively [58] and/or by a positive reduced adhesion formation after injury [61].

In the rbMixed secretome, in addition to the enriched molecular functions and biological processes mentioned above, the ECM relative processes such as “glycosaminoglycan (GAG) binding”, “proteoglycan biosynthetic process” and “ECM organization” (Figure 4E) were influenced. Glycosaminoglycans belong together with the proteoglycans to the non-collagen fraction of tendon ECM. Proteoglycans are a diverse group of ECM proteins, which regulate fibrillogenesis but are also involved in cell growth modulation and immune response stimulation [51]. The increased GAG binding may explain the enrichment ratio of sulfur compound binding, though enrichment was not significant (Figure 4A). In contrast to the upregulated protein fraction, no tendon or ECM-related functions or processes were enriched in the downregulated protein fraction (Figure 3B,F).

Although 182 of the approximately 2700 detected proteins showed a significant different expression between the two secretome groups (Figure 3B), most protein expression levels were similar (Figure S2D), including important ECM proteins such as COL I and COL III, with the latter playing an important role in tendon healing [53,58]. Notably, COL XIA1 was upregulated in the rbMixed secretome (Figure S2B) and thus contains a protein that is present in various tissues, including tendons, during development [62]. Compared to the produced secretomes from rabbit cells, the detected proteins were similar to those found in other studies using human donor cells [43] or human tenocytes additionally primed with biochemical cues [63].

The influence on the gene expression levels of tenocytes treated with secretome was examined in vitro for various markers of ECM remodeling and inflammation. Slight differences were observed between the rbMixed and rbADSC secretomes (Figure 5A,B). A strong upregulation of *IL-6* was detected for both secretomes but relative expression values of rbADSC secretome were significantly higher than for rbMixed secretome, which levels were not significantly higher compared to control cells at day 7 (Figure 5A). Although *IL-6* is defined as pro-inflammatory marker, it is a pleiotropic cytokine involved as well in the resolution of inflammation [64] and playing a crucial role in tendon healing [65]. Some studies reported that *COL I* expression was upregulated at the genetic level [66] and at the protein level in the presence of *IL-6* [67], while Katsma et al. [68] did not observe such an effect on the protein level. In our study, relative gene expression of *COL I* and *COL III* was downregulated significantly with both secretomes consistent with the results from Katsma et al. [68], who injected IL-6 into the Achilles peritendinous region of male Wistar rats showing that high *IL-6* levels suppressed gene expression of *COL I*. For secretome application, no changes of *COL I* on the protein level were observed in vitro analyzing cells from rats [69], whereas an upregulation of *COL I* [70] and *COL III* [71] was detected in a rat in vivo model. Besides the crucial role of *COL I* in tendons promoting biomechanical properties like stiffness, viscoelasticity and resistance to stress [53,58], *COL I* can be involved as well in adhesion formation, a process often occurring after tendon surgeries when scars extend from one tissue to another [72]. The genetic downregulation of *α-SMA*, *COL I* and *COL III* genes in the stimulated tenocytes in vitro caused by both secretomes might, therefore, reduce adhesion formation. The marker *α-SMA* is an inconsistent marker of contractile and collagen-producing fibroblasts, so-called myofibroblasts [72], which are widely believed to produce a high number of collagens upon excessive activity often leading to scar formation in consequence but playing a crucial role as well in wound contraction and closure upon tissue injuries. Besides *COL I* and *COL III*, tendon markers such as *TNMD* and *MKC* also were generally downregulated in the genetic level. No clear tendency for tendon marker *TNC* could be observed showing significantly higher values at day 1 with rbMixed secretome (1.7-times higher) and significantly reduced the gene expression level at day 3 with rbADSC secretome (Figure 5A). Although differences were observed in the gene expression level, it remains unclear how these changes would be reflected in the protein level as it has been reported that a downregulation of *COL I* in the genetic level was not observed in the protein level [69] in vitro.

No difference was detected between the two secretome groups for growth factors affecting tendon healing, such as VEGF, FGF-2, IGF-1 and PDGF-BB [73,74,75], and the proliferative effect of these growth factors was confirmed for both secretomes (Figure 6A). Similarly, tenocytes stimulated with one of the two secretomes showed significantly upregulated levels of *Ki67* gene expression in vitro at day 3 (2.4-times higher for rbADSC secretome and 3.6-times higher for rbMixed secretome compared to unstimulated cells; Figure 5B). This result is consistent with other studies reporting the positive effect of secretome on cell proliferation [76].

VEGF is one of the most important angiogenic factors regulating blood vessel formation in the early tendon healing phase [77]. While VEGF was expressed in both secretomes equivalently (Figure S2D), angiopoietin-1 (ANGPT1), playing an important role as well in vascular development and angiogenesis [78], showed higher protein expression levels in the rbMixed secretome (Figure S2B). The angiogenic effect of both secretomes is consistent with that reported in other studies [43,79].

The main growth factors upregulated in injured tendons, such as TGFβ, PDGF-BB [80] or typically VEGF, promote angiogenesis [81]. Angiogenesis is essential in the proliferative phase of tendon healing as it facilitates the healing process by delivering enough oxygen and nutrients, allowing the removal of waste products, transporting regulatory factors and controlling the immune response. Furthermore, new vessels are covered with pericytes, which represent a source of tendon/stem progenitor cells exhibiting MSC-like characteristics, such as self-renewal [77]. A proper angiogenesis during initial tendon healing is a prerequisite for a regenerative outcome as long as vessels retract in later phases. Patients with high VEGF levels in patellar tendinopathy healed faster than patients having low VEGF levels [82]. In addition, in the presence of VEGF, angiopoietin-1 stabilizes vessels and may be considered as a supporting factor for angiogenesis [83].

The two secretomes were not different with respect to their VEGF protein levels; however, there was more angiopoietin-1 protein detected in the rbMixed secretome. Once applied to an acute tendon injury, such angiopoietin-1 protein may, therefore, support initial angiogenesis when platelets secrete VEGF [80]. An earlier establishment of new vessels in turn may lead generally to a faster healing process with a higher tenoblast proliferation and a higher tendon/stem progenitor cell density due to earlier pericyte provision by newly formed vessels at the tendon wound site.

Ligand–receptor analysis revealed the differential upregulation of ligand–receptor pairs in the rbMixed secretome but not in the rbADSC secretome (Figure 3E), and the significantly enriched signaling pathways like Ras, Rap1, PI3K Akt, MAPK and HIF-1 (Figure 3F) known to influence proliferation, differentiation, cell growth, apoptosis and survival, inflammation, metabolism and angiogenesis, amongst other functions, were detected. Identified receptors and their ligand(s) were visualized in the paracrine network (Figure 3E). While some pairings might have relevant effects on tendon-healing influencing angiogenesis, cell survival, proliferation, migration and adhesion, pairings with VLDLR and Kremen do not seem to have direct beneficial effects on tendon healing but are involved in general developmental processes and in cell-to-cell and cell-to-matrix interactions. Neuropilin 2, a receptor for VEGF and PGF, is activating angiogenesis and endothelial cell growth, while the ligand semaphorin is involved in cardiovascular and central nervous system (CNS) development [84,85]. Angiopoietin-1 binds and activates the TEK receptor, also known as TIE2, playing an important role in the regulation of angiogenesis, endothelial cell survival, proliferation, migration and adhesion [78]. Angiopoietin-2 is another TEK ligand and able to modulate angiopoietin-1 signaling. In addition to the protein component analysis, secretomes were tested for their angiogenic function using the CAM assay. After 7 days, the CAM treated with the secretomes showed an increased formation of junctions (Figure 7B), increased vessel length (Figure 7D) and increased vessel density (Figure 7G), where treatment with rbMixed secretome showed an overall higher angiogenic activity index (Figure 7H).

It has been reported that IL-6 can induce VEGF production [64,77] and that treatment with secretome is able to induce VEGF synthesis as well [71]. Given that the rbMixed secretome contained receptor–ligand pairs that can have a beneficial effect on proliferation and migration, respectively, the wound closure of tenocytes under normal and inflammatory conditions after stimulation with the respective secretome was examined in vitro using the migration assay. The LPS used to mimic an inflammatory environment causes a 10-fold increase in gene expression compared to untreated cells [86]. Wound closure was less advanced under LPS conditions and after secretome treatment than under normal conditions (Figure 6B,C). This was also observed in cultures where no secretome was added [87], but treatment with secretome, especially with that derived from rbADSCs, may boost the effect [88] in both conditions (Figure 6B–G).

Overall, a positive effect of secretome on tendon healing was reported [37,40,71], although a comparison and interpretation of the published results remain difficult as donors from different species were used, the production of secretomes varied, the results were obtained from in vitro or in vivo experiments, respectively, and experimental protocols were not identical. To gain more information about the effects of the secretomes produced and to verify the promising in vitro results, their application in a rabbit full laceration model is envisaged [89]. For this purpose, a combination with an established electrospun biomaterial consisting of DegraPol^®^ polymer [90], which has been used for growth factor delivery to improve tendon healing [14,56,91], is intended to be applied. Regarding the translation of the secretome treatment of tendons into medicine in the future, secretome production with ADSCs might be favorable due to the simple harvest of patient cells [92]. However, in contrast to the proteomic profile of rbADSC secretome, rbMixed secretome was convincing in terms of molecular functions and biological processes associated with ECM and neovascularization, potentially beneficial for tendon healing processes. This novel approach could pave the way for a cell-free therapeutic strategy for tendon injuries by targeting paracrine mechanisms.

Some limitations of the study need to be mentioned. First, we did not examine the secretome of tenocytes alone. Due to the low proliferation and passaging rate of the cells, the feasibility of producing secretome was severely limited by the scope of this study. Secondly, gene expression analyses were not performed on tenocytes that had been exposed to an inflammatory environment. This may result in an altered gene expression profile in the in vivo situation. Furthermore, the key proteins identified in the proteomic analysis were not used as single factors in this study. This is of great interest, and a further investigation is planned to examine the effects on the ECM and to compare the efficacy of the secretome. Finally, the efficacy of the secretome and its influence on the healing process and on the biomechanical properties of the treated tendons must be further investigated. In the in vitro model, the examination of these aspects is limited or entirely impossible, which explains the need for a corresponding in vivo study. This will allow the observed findings to be linked to the determined tissue mechanics and function, which can be comprehensively studied using the isolated tissue using the rabbit Achilles tendon full transection model [89].

## 4. Materials and Methods

### 4.1. Isolation of Rabbit-Adipose-Derived Mesenchymal Stem Cells

Rabbit adipose tissue from the kidney region of three New Zealand white rabbits (females, 5- to 7-month-old) was provided by the University Hospital of Zurich, Switzerland (ethical approval No. 255/15, veterinary office of Canton Zurich, Zurich, Switzerland). Rabbit ADSCs were isolated according to standard protocols as described elsewhere [93]. Briefly, adipose tissue (1.6 ± 1.7 g) was washed with Dulbecco’s phosphate-buffered saline (PBS; Merck/Sigma-Aldrich, Buchs, Switzerland) containing 100 U/mL penicillin, 100 µg/mL streptomycin (Thermo Fisher Scientific, Schlieren, Switzerland) and 2.5 µg/mL amphotericin B (Pan Biotech, Aidenbach, Germany) and minced using two scalpels or scissors. The adipose tissue was digested at 37 °C for up to 1 h in Dulbecco’s modified eagle’s medium with high glucose (DMEM-HG; Merck/Sigma-Aldrich, Buchs, Switzerland) containing 1 mg/mL collagenase type A (Roche, Basel, Switzerland), 2 mM glutamax-I (Thermo Fisher Scientific, Schlieren, Switzerland). The same concentration of penicillin, streptomycin, glutamax-I and amphotericin B (P/S/G/A) as before, together with 10% heat inactivated fetal calf serum (FCS; Biowest, Nuaillé, France), was added to DMEM-HG and used to dilute the enzymatic solution at the end of the incubation period. The digested tissue was filtrated using a 70 µm cell strainer and centrifugated at 400× *g* for 10 min at room temperature. After aspirating the supernatant, the cell pellet was resuspended in DMEM-HG + 1% P/S/G/A + 10% FCS, transferred to 10 cm petri dishes and cultured at 37 °C and 5% CO_2_. The culture medium was replaced every 3–4 days.

### 4.2. Isolation of Rabbit Achilles Tenocytes

The Achilles tendons were provided by the University Hospital Zurich, Zurich, Switzerland (approval by the veterinary office of Canton Zurich, Zurich, Switzerland; reference number 255/15). The Achilles tendons of New Zealand white rabbits (*n* = 6, females, 5 to 7 months old) were isolated according to standard protocols as described elsewhere [86]. Briefly, the tendons were washed in PBS with 200 µg/mL gentamicin sulfate (Biowest, Nuaillé, France) and 2.5 µg/mL amphotericin B. Surrounding tissue was removed using a scalpel and a tweezer. The center part of each tendon was washed three times in the PBS before transferring it into a 10 cm petri dish. The tendons were cut into very small pieces (<1 mm) using two scalpels. The cell mass was spread out regularly onto the Petri dish, and 10 mL Ham’s F12 (Biowest, Nuaillé, France) containing 10% FCS and P/S/G was added. The tissue was incubated at 37 °C and 5% CO_2_ for 5 days without moving the plate to allow the cells migrating out of the tissue. Culture medium was changed every third day, and, after approximately 10 days, the tissue pieces were removed from the plate. Tenocytes were transferred into cell culture flasks and allowed to grow further before they were deep-frozen in liquid nitrogen. To thaw cryopreserved tenocytes, they were resuspended in culture medium (Ham’s F12, 10% FCS with 1% P/S/G) and cultured at 37 °C and 5% CO_2_. The medium was changed every second day.

### 4.3. Phenotypical Characterization of Rabbit-Adipose-Derived Mesenchymal Stem Cells and Rabbit Achilles Tenocytes

Lineage differentiation of rbADSCs into osteocytes, adipocytes and chondrocytes was examined as previously described [94]. Briefly, rbADSCs of three donors were cultured in osteogenic medium consisting of DMEM low glucose (Merck/Sigma-Aldrich, Buchs, Switzerland), 10% FCS, 10 mM b-glycerophosphate (Merck/Sigma-Aldrich, Buchs, Switzerland), 50 mM L-ascorbic acid 2-phosphate (Merck/Sigma-Aldrich, Buchs, Switzerland), 100 nM dexamethasone (Merck/Sigma-Aldrich, Buchs, Switzerland) and 1% P/S. After three weeks of cultivation with a medium change twice a week, the cells were fixed with 4% formalin (Formafix Switzerland AG, Hittnau, Switzerland). A solution of 2% alizarin red (Merck/Sigma-Aldrich, Buchs, Switzerland) was used to stain calcium deposits. The induction of adipogenic differentiation was initiated by incubation with DMEM low glucose, 10% FCS, 1 mM dexamethasone, 10 mg/mL insulin (Merck/Sigma-Aldrich, Buchs, Switzerland), 120 mM indomethacin (Merck/Sigma-Aldrich, Buchs, Switzerland), 0.5 mM 3-isobutyl-1-methylxanthine (IBMX; Merck/Sigma-Aldrich, Buchs, Switzerland) and 1% P/S for three days. The cells were then kept in maintenance medium, which corresponds to the induction medium without IBMX supplementation, for 18 days with regular medium changes twice a week. After fixation with 4% formalin, the lipid vacuoles were stained with 3% oil red-o solution (Merck/Sigma-Aldrich, Buchs, Switzerland). Chondrogenic differentiation was induced in cell aggregates using DMEM-HG supplemented with 1% FCS, 0.5 mg/mL insulin, 50 mM L-ascorbic acid-2-phosphate, 10 ng/mL TGFβ1 (PeproTech, London, UK) and 1% P/S. After a cultivation period of three weeks with two medium changes per week, the aggregates were fixed in 4% formalin, embedded in paraffin and sectioned. To detect proteoglycans, the sections were stained with 0.1% toluidine blue solution (Merck/Sigma-Aldrich, Buchs, Switzerland). Microscopic documentation was carried out using a Zeiss Axio Vert.A1 brightfield microscope with ZEN 2.6 lite software (Carl Zeiss Microscopy, Oberkochen, Germany). Both cell types, rbADSCs and rbTenocytes (three donors each), were characterized by quantitative RT-PCR based on gene expression profile of the following rabbit-specific markers (Table 1) *CD34*, *CD44*, *CD45*, *CD90*, *CD105*, *collagen 1A2*, *collagen 3A1*, *mohawk*, *tenascin C* and *tenomodulin*. The housekeeping gene *GAPDH* was used to quantify relative cell expression using the −ΔCT threshold cycle method. The analysis was performed twice with technical triplicates in each case. The procedure was analogous to the RT-PCR method described in the next subchapter called evaluation of gene expression by quantitative RT-PCR.

### 4.4. Production of Two Types of Secretome

In order to have a common medium for both cell types, the cells were cultivated after the first passage in homemade medium consisting of complete medium for the rbADSCs and complete medium for rbTenocytes. Specifically, DMEM-HG + 10% FCS + 1% P/S/G was prepared with Ham’s F12 + 10% FCS + 1% P/S/G in a 1:1 ratio. Cells were maintained either as a pure rbADSC culture or as a mixed culture consisting of rbADSCs and rbTenocytes in a 3:1 ratio (rbMixed). To minimize the influence of possible inter-donor variability, three donors per cell type were pooled in culture for secretome preparation. The cells were allowed to grow at 37 °C and 5% CO_2_ to 80% confluence in complete medium. The cells were then thoroughly washed three times with PBS, and serum-free culture medium (SFM) was added. After 48 h of cultivation, the supernatant was removed, and the two different secretomes obtained were centrifuged at 4 °C for 10 min at 2200 rpm to separate possible cell debris. The two secretomes were concentrated 10-fold at 4 °C using a 3 kDa cut-off centrifugal concentrator (Millipore/Merck, Buchs, Switzerland). After aliquoting, the samples were stored at −80 °C. The viability of the starved cells was determined using a NucleoCounter NC-200 (ChemoMetec, Lillerød, Denmark).

### 4.5. Characterization of Secretomes by Determination of Total Protein Content and Proteomics Analysis

Protein concentration of the different harvests was determined with DC Protein Assay Kit (Bio-Rad, Cressier, Switzerland) according to manufacturer’s protocol. Secretomes containing 1% P/S/G without FCS were concentrated 10 times before analysis. Experiment was carried out in technical quadruple, and bovine serum albumin (BSA; Sigma-Aldrich/Merck, Buchs, Switzerland) diluted in culture medium without FCS was used for standard curve. Proteomics was performed at the Functional Genomics Center Zurich (FGCZ) of University of Zurich, Zurich, Switzerland, and ETH Zurich, Zurich, Switzerland. Measurements were carried out in technical triplicates using 1000 µL FCS-free secretome with a protein concentration of 0.04 µg/µL. Briefly, the secretomes were dried down to approximately 150 µL and lysed with 4% sodium dodecyl sulfate (SDS; Sigma-Aldrich/Merck, Buchs, Switzerland) for 10 min at 95 °C. Then tris(2-carboxyethyl)phosphine (Sigma-Aldrich/Merck, Buchs, Switzerland) and 2-chloroacetamide (Sigma-Aldrich/Merck, Buchs, Switzerland) were added to a final concentration of 5 mM and 15 mM before secretomes were incubated for 30 min at 30 °C under shaking conditions at 700 rpm and protected from light. Samples were diluted with pure ethanol to a final concentration of 60% (*v*/*v*) before the corresponding amount of carboxylated magnetic beads (hydrophobic and hydrophilic) were added using the KingFisher Flex System (Thermo Fisher Scientific, Schlieren, Switzerland). Proteins were allowed to bind to the beads for 30 min at RT before beads were washed 3 times with 80% ethanol. Samples were digested over night at 37 °C in 50 mM triethylammonium bicarbonate (TEAB; Sigma-Aldrich/Merck, Buchs, Switzerland) containing trypsin (Sigma-Aldrich/Merck, Buchs, Switzerland) in a trypsin protein ratio 1:50. Remaining peptides were extracted from beads with water, and the two elutions were combined and dried down. For LC–MS/MS, the digested samples were dissolved in aqueous 3% acetonitrile (Sigma-Aldrich/Merck, Buchs, Switzerland) with 0.1% formic acid (Sigma-Aldrich/Merck, Buchs, Switzerland) before peptide concentration was estimated with the Lunatic UV/Vis absorbance spectrometer (Unchained Labs, Pleasanton, CA, USA). Samples were loaded on evotips (Evosep, Odense, Denmark) according to the provided instructions and analyzed on an Evosep One LC coupled to a TIMS TOF Pro mass spectrometer (Bruker, Ettlingen, Germany). The acquired data independent acquisition spectra were processed with DIA-NN (Version 16, PMID: 31768060) using a library free approach with the following protein database(s): fasta/fgcz_9986_1spg_rabbit_20230615. Variable modifications such as acetyl (protein N-term), oxidation (M), met-loss (protein N-terminal) and fixed modification like carbamidomethyl (C) were set. Differential expression analysis was performed to verify that the difference between normalized empirical protein abundances measured in two groups is significantly non-zero. A set of functions implemented in the R-Package prolfqa (running R version 4.1.2) [96] were run to filter and normalize the data, generate visualizations and compute differential expression analysis. The false discovery rate (FDR) was computed using the Benjamini–Hochberg (BH) procedure, and proteins were filtered using a FDR threshold of 10% [97]. Fold change of the differentially expressed proteins was set at minus and plus 1. To run over-representation analysis (ORA), human homologies of the rabbit proteins were determined, and WEB-based GEne SeT AnaLysis Toolkit [49] was used. Visual presentation of the data was performed with GraphPad Prism 9 for Windows (GraphPad Software, San Diego, CA, USA).

### 4.6. Ligand–Receptor Interaction Analysis and Functional Enrichment

We used a curated ligand–receptor interaction database [47] to programmatically identify secreted ligands and their corresponding receptors. Upregulated and downregulated transcripts were processed separately from the differential expression analysis data. Ligand and receptor gene symbols were compared to a predefined list of expressed genes to extract potential paracrine interactions. Only ligand–receptor pairs where both components were detected in the dataset were considered.

### 4.7. Evaluation of Gene Expression by Quantitative RT-PCR

To determine the effect of the two differently produced secretomes on the gene expression of tenocytes in vitro, rabbit tenocytes were isolated and cultured as described in the previous section. Cells were seeded into 12-well plates (Sigma-Aldrich/Merck, Buchs, Switzerland, growth area per well: 3.5 cm^2^) with a density of 1 × 10^5^ cells/well in 1 mL culture medium. Cells were allowed to attach overnight before culture medium was exchanged to fresh culture medium or secretomes both supplemented with 10% FCS + 1% P/S/G. Medium and secretomes were exchanged on days 0, 2, 4 and 6, and tenocytes were collected at days 1, 3 and 7 for further RNA extraction. Rabbit tenocytes from four different animals were used (n = 4) and cultured in duplicates. Expression levels were analyzed in technical triplicates.

Total RNA was extracted using RNeasy Mini Kits (Qiagen, Zug, Switzerland), and reverse transcription was carried out using Superscript III RT (Invitrogen/Thermo Fisher Scientific, Schlieren, Switzerland) according to the manufacturer’s instructions. Amount and purity of RNA were measured with Nanodrop One (Thermo Fisher Scientific, Schlieren, Switzerland). For reverse transcription, 500 ng RNA was investigated in a reaction volume of 20 µL SuperScript III Reverse Transcriptase (Thermo Fisher Scientific, Schlieren, Switzerland), Oligo(dT)12-18 Primer (Thermo Fisher Scientific, Schlieren, Switzerland), RNase inhibitor (Applied Biosystem/Thermo Fisher Scientific, Schlieren, Switzerland) and dNTP (Invitrogen/Thermo Fisher Scientific, Schlieren, Switzerland) using a compact thermocycler (Masterscycler personal, Eppendorf, Germany). Quantitative real-time PCR reactions were performed with 4 µL of the resulting cDNA using the Quant Studio 5 (Applied Biosystems/Thermo Fisher Scientific, Schlieren, Switzerland), rabbit-cell-specific primers (Microsynth, Balgach, Switzerland; Table 1) and Fast SYBR Green Master Mix (Thermo Fisher Scientific, Schlieren, Switzerland). Samples were heated to 95 °C for 3 min, followed by 40 cycles of 95 °C for 3 s and 60 °C for 20 s. The gene 18S served as housekeeping gene to quantify relative expression levels after tenocyte stimulation using the ΔCT threshold cycle method. Results were analyzed with RStudio 2023.12.1 using the linear mixed model with fold change as response variable, medium type as fixed variable and rabbit as intercept for all genes. *p*-values were adjusted using Benjamini–Hochberg correction.

### 4.8. Determination of Metabolic Activity Using alamarBlue Assay

Cell viability was determined on days 3, 5, 7 and 10 using alamarBlue cell viability assay (Invitrogen/Thermo Fisher Scientific, Schlieren, Switzerland). For experiment, 350 cells/well in technical triplicates were transferred into a 96-well plate (Techno Plastics Products AG, Trasadingen, Switzerland) in 100 µL culture medium DMEM-HG + 10% FCS + 1% P/S/G with Ham’s F12 + 10% FCS + 1% P/S/G in a 1:1 ratio or in 10× concentrated secretome supplemented with 10% FCS + 1% P/S/G and cultured at 37 °C with 5% CO_2_. Empty wells were filled with PBS to prevent dehydration. The alamarBlue solution was diluted 1:10 in cell culture medium, cells were incubated with 100 µL alamarBlue solution for 4 h before excitation wavelength of 530 nm and emission wavelength of 590 nm was measured using a Cytation 5 imaging reader (BioTEk, Agilent Technologies AG, Basel, Switzerland). After measurement, cells were washed once with cell culture medium before 100 µL culture medium or secretome was added for further culture. Medium and secretome was exchanged every second day on days 0, 2, 4, 6 and 8.

### 4.9. Determination of Anti-Inflammatory Capacity In Vitro Using Tenocyte-Based Scratch Assay

Rabbit tenocytes were grown to confluence in culture medium in 48-well plate. The addition of 100 ng/mL lipopolysaccharide (LPS; Merck/Sigma-Aldrich, Buchs, Switzerland) was used to mimic an inflammatory condition. Cells were treated with or without LPS for 24 h, and the cell monolayer was then incised with a tip of a 200 μL pipette. The well was then washed twice with PBS to remove any loose cells. After the addition of either secretome based on pure rbADSC culture or secretome based on the mixed culture of rbADSCs and rbTenocytes or medium as control, the remaining cells were cultured with or without LPS for 24 h under standard conditions. To ensure an optimal supply of nutrients, FCS was added to all three groups at a final concentration of 10%. Images of scratched monolayers were obtained at 0, 6 and 24 h. The size of the wounds was measured by digital morphometric analysis. The reduction in the area of the scratch after cultivation corresponds inversely proportional to the percentage of wound closure. In addition, the width of the scratch was measured at five different positions distributed over the image and evaluated as a second readout for wound healing in vitro. The calculated percentages refer to the reduction in wound width over time. Cells were examined and images were generated with the Zeiss Axio Vert.A1 brightfield microscope and ZEN 2.6 lite software (Carl Zeiss Microscopy, Oberkochen Germany). A total of eight approaches with three independent rbTenocytes donors were performed and analyzed for each condition.

### 4.10. Evaluation of Angiogenic Potential of Secretomes In Ovo

According to the Swiss animal welfare guidelines (TSchV, Art. 112), no IACUC approval is required until the 14th day of development of the chicken embryo. White LSL chicken eggs from Lohmann (Animalco AG Geflügelzucht, Staufen, Switzerland) were incubated for 4 days at 37 °C and 50–70% humidity. Afterwards, the shell was fenestrated to carefully open the egg. In addition, 5 mL of albumin was taken per egg to ensure that the chorioallantoic membrane (CAM) could be lowered. The opening was sealed under sterile conditions with a commercially available breathable patch and incubated for a further three days. On the seventh embryonic development day (EDD7), a plastic ring was positioned on the CAM to limit the CAM region to be treated before application of the respective secretome group or medium control group (*n* = 17 per group). From EDD7 to EDD14, 30 μL of the solution to be examined (secretomes or medium control) was applied every second day (EDD7 = d0, EDD9 = d2, EDD11 = d4 and EDD13 = d6). The CAM was microscoped and photographed at the beginning of the treatment (EDD7) and on days EDD9, EDD11 and EDD14. On EDD14, seven days after treatment of the CAM, the CAM was fixed using 4% formalin (Formafix, Hittnau, Switzerland) and histologically processed. After paraffin embedding, 3 μm sections were cut and stained with H&E. Imaging was performed with equipment maintained by the Center for Microscopy and Image Analysis, University of Zurich (Zurich, Switzerland). The comparison of the two secretome types was evaluated by determining a group of different parameters selected based on probability of survival, number of junctions, total vessel length, mean vessel length, vessel density, branch hierarchy and CAM thickness. All parameters were measured manually using ImageJ software (version 2.9.0, NIH, Bethesda, MD, USA) and QuPath software (v0.5.1, University of Edinburgh, Edinburgh, UK) [98]. Based on the values obtained on days 0, 2, 4 and 7, the angiogenic profile was generated, and the angiogenic activity index (AAI) was calculated as described earlier [50,99]. In order to take into account the vascularization present at the beginning of treatment, which is individual for each embryo, the fold change was calculated by dividing the values obtained on treatment days 2, 4 and 7 by the value obtained before the start of treatment (day 0). Branch hierarchy was used to calculate the AAI as the ratio of 3rd- and 4th-degree branches to 1st- and 2nd-degree branches to reflect the extent of a higher hierarchy of vascular network structure in the AAI. The AAIs were determined separately for each parameter summarized as follows as example for day 7 (Equation (1)).

(1) $$\mathrm{AAI}\;\mathrm{for}\;\mathrm{parameters}=\;\frac{\left\{{AR}^{7thDoT}\left[Treatment\right]-{AR}^{0DoT}\left[Treatment\right]\right\}-\left\{{AR}^{7thDoT}\left[Control\right]-{AR}^{0DoT}\left[Control\right]\right\}}{\left\{{AR}^{7thDoT}\left[Control\right]-{AR}^{0DoT}\left[Control\right]\right\}}$$

where AR represents the angiogenic response of the respective examined parameter, and DoT represents the day of treatment with the secretome groups (Treatment) or medium group (Control). The final AAI was calculated based on the mean of the parameters (Equation (2)):

(2) $$\mathrm{Final}\;\mathrm{AAI}=\frac{1}{n}\sum_{i=1}^{n}{AAI}_{i}$$

where *n* is the number of parameters, and AAI_i_ denotes the individual values of AAI.

### 4.11. Statistical Evaluation

Data in the text are represented as means ± standard deviations (SD) unless otherwise stated. The ROUT test was used to identify possible outliers, which were then excluded from further analyses. The Shapiro–Wilk test was applied to analyze normality of the data distribution. Comparisons between groups were performed with Kruskal–Wallis test followed by Dunn’s multiple comparison test or with Mann–Whitney test. *p*-value adjustments were carried out with Benjamini–Hochberg correction. Statistical analysis was performed to compare groups using the log-rank test (Mantel–Cox) when determining the probability of survival for the CAM assay. Migration and CAM assay data were compared using a repeated measures two-way ANOVA (mixed model) with a Geisser–Greenhouse correction and Tukey’s post hoc correction for multiple comparisons. A *p* value < 0.05 was considered statistically significant. Data were analyzed and visualized with Microsoft Excel (version 16.77, USA) and GraphPad Prism software (version 10.0.2, USA).

## 5. Conclusions

Two different types of secretomes were produced, either by using rbADSCs only or by a combination of rbADSCs and rbTenocytes (rbMixed), with the aim of studying and comparing their effects in vitro and in ovo and to generate a proteomic profile. The results will allow us to assess which secretome is more potent in terms of tendon healing and could be used in the future in a rabbit full laceration Achilles tendon model. The analysis of the LC–MS/MS data showed that the two secretomes were clearly distinguishable, with 182 proteins being significantly different in expression. Compared to the rbADSC secretome, the upregulated protein fraction of the rbMixed secretome showed increased levels of tendon-related proteins like BGN and TNC. Most proteins were equally expressed, including growth factors important for tendon healing such as FGF-2, VEGF, IGF-1 and PDGF-BB, which might explain similar outcomes regarding the increased proliferation rate and improved angiogenesis. The rbADSC secretome demonstrated a superior wound closure under both healthy and inflammatory conditions. The relative gene expression levels of tenocytes treated with both secretomes showed similar alterations compared to control cells in medium, with some differences. Like for *IL-6*, an increase was also observed for ECM remodeling factors *MMP-9* and *Timp1*. Other ECM relevant factors like *COL I*, *COL III* and *α-SMA* or tendon markers such as *TNMD* and *MKX* were downregulated when both secretomes were applied. Our study demonstrates the potential of secretome to influence cellular processes and improve healing. According to our results, the treatment of tendon injuries with rbMixed secretome might be more beneficial compared to rbADSC secretome, but further studies and translation into an in vivo model are required to substantiate this conclusion.

## Figures and Tables

**Table 1:** Primer sequences for quantitative real-time polymerase chain reaction. Forward and reverse primers used for gene expression analysis.

Gene	Primer Sequence	GenBank Accession No.	Reference
*18s-rRNA*	Forward *5′* CGG AAC TAC GAC GGT ATC TG *3′* Reverse 3′ *GGA ACT GAG GCC ATG ATT AAG 5′*	NR_033238.1	[86]
Rabbit *ACTA2*	Forward *5′* CGT GAC TAC TGC TGA ACG TG *3′* Reverse 3′ *GGA TGC CAG CAG ATT CCA TC 5′*	NM_001101682.2	[86]
Rabbit *ALOX15*	Forward *5′* CGA CTT CGC CCT GCT GGA TA *3′* Reverse 3′ *GCT GGA TGA CCA TAG GCA TGA 5′*	NM_001082282.1	[86]
Rabbit *BGN*	Forward *5′* GGC CTG AAG CTC AAC TAC CT *3′* Reverse 3′ *GGC TCC CGT TCT CAA TCA TC 5′*	NM_001195691.1	[86]
Rabbit *CD34*	Forward *5′* CTG AGG TTA GGG CTC AGT GC *3′* Reverse 3′ *GGA GTA GCT CTG GTG GCT TG 5′*	XM_008268472.3	[95]
Rabbit *CD44*	Forward 5′ TCA TCC TGG CAT CCC TCT TG 3′ Reverse 3′ CCG TTG CCA TTG TTG ATC AC 5′	XM_008269711.2	[95]
Rabbit *CD45*	Forward 5′ TAC TCT GCC TCC CGT TG 3′ Reverse 3′ GCT GAG TGT CTG CGT GTC 5′	XM_008268695.3	[95]
Rabbit *CD90*	Forward 5′ CTG CTG CTC TCA CTG TC 3′ Reverse 3′ ACA GAA GCA GCT TTG GGA AA 5′	XM_002722718.4	[95]
Rabbit *CD105*	Forward 5′ TGA CAT ACA GCA CCA GCC AG 3′ Reverse 3′ AGC TCT GAC ACC TCG TTT GG 5′	XM_008251029.3	[95]
Rabbit *COL1A1*	Forward 5′ CTG GTG AAT CTG GAC GTG AG 3′ Reverse 3′ TGT CTC ACC CTT GTC ACC AC 5′	XM_008271783.1	[86]
Rabbit *COL3A1*	Forward 5′ GCA TTG CTT ACA TGG ATC AGG 3′ Reverse 3′ CCA ACG TCC GCA CCA AAT TC 5′	XM_008254949.1	[86]
Rabbit *GAPDH*	Forward 5′ CAA GAA GGT GGT GAA GCA GG 3′ Reverse 3′ GCT GTA GCC AAA TTC GTT GTC 5′	NM_001082253.1	-
Rabbit *IL-6*	Forward 5′ GAA AAC ACC AGG GTC AGC AT 3′ Reverse 3′ CAG CCA CTG GTT TTT CTG CT 5′	AF169176	[86]
Rabbit *MKI67*	Forward 5′ CAC ATC CAG CAG TGA AAC GG 3′ Reverse 3′ GTG TTA GCA GTA CCT GAA GTC 5′	XM_008251084.2	[86]
Rabbit *MMP-2*	Forward 5′ GAA GAT CGA CGC TGT GTA CG 3′ Reverse 3′ GTA TCT CCA GAA CTT GTC TCC 5′	D63579.1	[86]
Rabbit *MMP-9*	Forward 5′ GAT ACA GCC TGT TCC TCG TG 3′ Reverse 3′ GGA CCA TAT AGA TGC TGG ATG 5′	D26514.1	[86]
Rabbit *MKX*	Forward 5′ AAC GTG GAG CAG TCT CTG AG 3′ Reverse 3′ CAC GCA CTC TGG TAC AGT TG 5′	NC_013684.1	[86]
Rabbit *TNC*	Forward 5′ GTC ACT CAT CAC AGC TCT GG 3′ Reverse 3′ CTG AGT GTG TAT TCC GTG GC 5′	FJ480400.1	[86]
Rabbit *TNMD*	Forward 5′ GCA GTT TCC GAG TTA CAA GAC 3′ Reverse 3′ CGA CGG CAG TAA ATA CAA CAG 5′	NM_001109818.1	[86]
Rabbit *TIMP1*	Forward 5′ CTA CCT TGT ACC AGC GTT ATG 3′ Reverse 3′ GAA GCT CAG ACT GTT CCA GG 5′	NM_001082232.2	[86]

Gene abbreviations: *18s-rRNA*: 18S ribosomal RNA, *ACTA2*: alpha-smooth muscle actin, *ALOX15*: arachidonate 15-lipoxygenase, *BGN*: biglycan, *CD*: cluster of differentiation, *COL1A1*: collagen type I alpha 1 chain, *COL3A1*: collagen type III alpha 1 chain, *GAPDH*: glyceraldehyde-3-phosphate dehydrogenase, *IL*: interleukin, *MKI67*: marker of proliferation Ki-67, *MMP*: matrix metalloproteinase, *MKX*: mohawk homeobox, *TNC*: tenascin-C, *TNMD*: tenomodulin and *TIMP1*: TIMP metallopeptidase inhibitor 1.

## Data Availability

Data is contained within the article and Supplementary Materials.

## Supplementary Materials

The following supporting information can be downloaded at https://www.mdpi.com/article/10.3390/ijms26083622/s1.
