# Supplementary material for: Therapeutic Potential of Mesenchymal Stem Cell and Tenocyte Secretomes for Tendon Repair: Proteomic Profiling and Functional Characterization In Vitro and In Ovo
Source: Int J Mol Sci. 2025 Apr 11;26(8):3622. doi: 10.3390/ijms26083622 (PMC12026810; doi:10.3390/ijms26083622)
Supplement: Supplementary file 1 [file ijms-26-03622-s001.zip › ijms-3553455-supplementary.pdf]

Supplementary Materials

Therapeutic potential of mesenchymal stem cell and tenocyte secretomes for tendon repair: proteomic profiling and functional characterization *in vitro* and *in ovo*

Petra Wolint [1#], Iris Miescher [1#], Asma Mechakra [2,3], Patrick Jäger [2,3], Julia Rieber [1], Maurizio Calcagni [1], Pietro Giovanoli [1], Viola Vogel [4], Jess G Snedeker [2,3] and Johanna Buschmann [1*]

[1] Division of Plastic Surgery and Hand Surgery, University Hospital Zurich, 8091 Zurich, Switzerland
[2] Institute for Biomechanics, ETH Zurich, 8092 Zurich, Switzerland
[3] Balgrist University Hospital, University of Zurich, 8008 Zurich, Switzerland
[4] Laboratory of Applied Mechanobiology, Department of Health Sciences and Technology, ETH Zurich, 8092 Zurich, Switzerland
[#] Shared first authorship
[*] Correspondence: Johanna Buschmann, Plastic Surgery and Hand Surgery, University Hospital of Zurich, Raemistrasse 100, 8091 Zurich, Switzerland

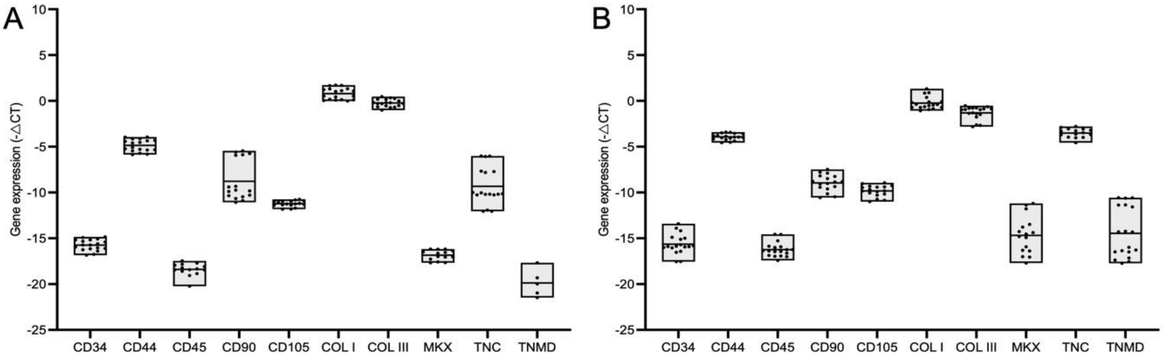

	CD34	CD44	CD45	CD90	CD105	COL I	COL III	MKX	TNC	TNMD
CD34		***	ns	ns	ns	***	***	ns	ns	ns
CD44	***		***	ns	ns	ns	ns	***	ns	***
CD45	ns	***		***	ns	***	***	ns	***	ns
CD90	ns	ns	***		ns	**	ns	*	ns	*
CD105	ns	ns	ns	ns		***	***	ns	ns	ns
COL I	***	ns	***	**	***		ns	***	**	***
COL III	***	ns	***	ns	***	ns		***	*	***
MKX	ns	***	ns	*	ns	***	***		*	ns
TNC	ns	ns	***	ns	ns	**	*	*		*
TNMD	ns	***	ns	*	ns	***	***	ns	*	

	CD34	CD44	CD45	CD90	CD105	COL I	COL III	MKX	TNC	TNMD
CD34		***	ns	*	ns	***	***	ns	***	ns
CD44	***		***	ns	ns	ns	ns	***	ns	***
CD45	ns	***		**	*	***	***	ns	***	ns
CD90	*	ns	**		ns	**	*	ns	ns	ns
CD105	ns	ns	*	ns		***	**	ns	ns	ns
COL I	***	ns	***	**	***		ns	***	ns	***
COL III	***	ns	***	*	**	ns		***	ns	***
MKX	ns	***	ns	ns	ns	***	***		***	ns
TNC	***	ns	***	ns	ns	ns	ns	***		***
TNMD	ns	***	ns	ns	ns	***	***	ns	***	

Figure S1. Characterization of rabbit adipose tissue-derived mesenchymal stem cells (rbADSCs) and rabbit tenocytes (rbTenocytes) using qPCR. Gene expression of the isolated and cultured rbADSCs was determined for the following markers *CD34*, *CD44*, *CD45*, *CD90*, *CD105*, *COL I*, *COL III*, *MKX*, *TNC*, and *TNMD* and normalization was performed using the -deltaCT method (A). The same marker set was determined for the characterization of the isolated and cultured rbTenocytes (B). Markers were compared using nonparametric Kruskal-Wallis test (not significant (ns); $*p < 0.05$; $**p < 0.01$; $***p < 0.001$).

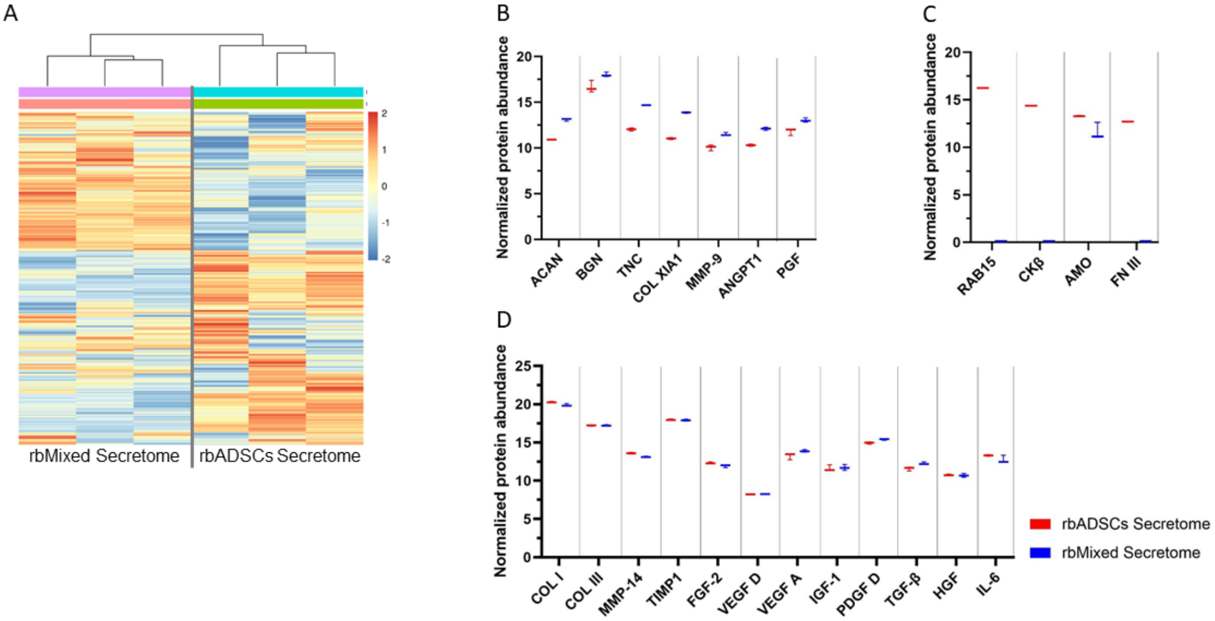

Figure S2. Protein expression detected in secretomes. Protein abundance heatmap (rows indicate proteins, columns indicate samples) showing the row scaled log_2 transformed protein abundance value (A). Selection of protein expression based on normalized protein abundance showing upregulated proteins in rbMixed secretome (secretome obtained from rbADSCs and rbTenocytes culture (ratio 3:1); B), downregulated proteins in rbMixed secretome (C), and equally expressed proteins in both secretomes, rbADSC and rbMixed (D).

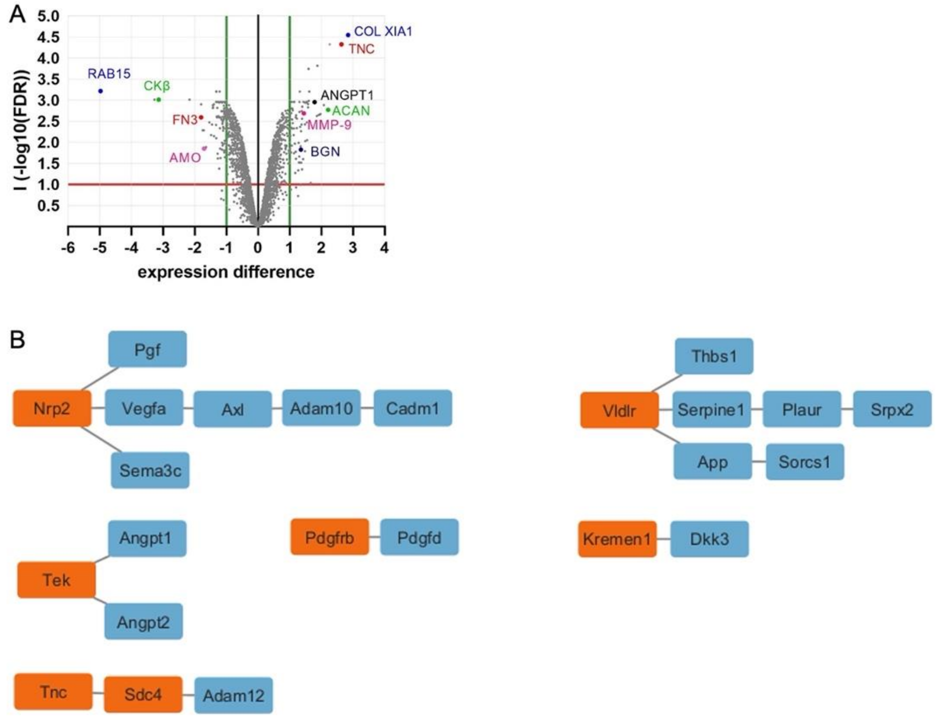

Figure S3. Volcano plot and paracrine network analysis of secretomes. Volcano plot with −log10 transformed FDR as function of the protein expression difference be-tween groups (A). The red line indicates the −log10(FDR) of FDR = 0.05, while the green lines rep-resent the difference of minus and plus 1. Selection of differently expressed proteins is highlighted. Paracrine network of ligand-receptor pairs of rbMixed secretome (B). Receptors are highlighted in

orange and ligands in blue color. Abbreviations: Adam10 = a disintegrin and metalloproteinase domain containing protein 10, Adam12 = a disintegrin and metalloproteinase domain containing protein 12, Angpt1 = angiopoietin 1, Angpt2 = angiopoietin 2, App = amyloid precursor protein, Axl = AXL receptor tyrosine kinase, Cadm1 = cell adhesion molecule 1, Dkk3 = dickkopf 3, Nrp2 = neuropilin 2, Pdg-frb = plateled-derived growth factor receptor β, Pdgfd = plateled-derived growth factor D, Pgf = placental growth factor, Plaur = plasminogen activator, urokinase receptor, Sdc4 = syndecan 4, Sema3c = semaphorin 3c, Sorcs1 = sortilin related VPS10 domain containing receptor 1, Srpx2 = sushi repeat containing protein X linked 2, Tek = TEK receptor tyrosine kinase, Thbs1 = thrombospondin 1, Tnc = tenascin-C, Vegfa = vascular endothelial growth factor A, Vldlr = very low density lipoprotein receptor.

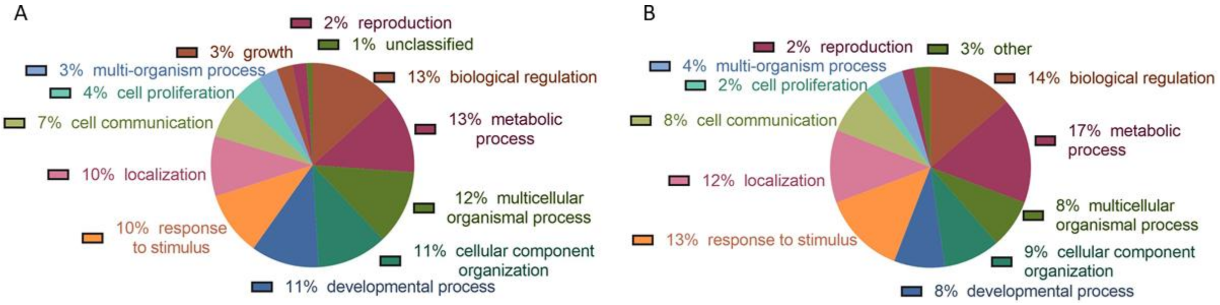

Figure S4. Enrichment analysis of secretomes. Activities of biological processes of upregulated proteins (A) and of downregulated proteins (B) in rbMixed secretome (rbADSCs and rbTenocytes culture in a ratio 3:1) compared to rbADSC secretome. Parameters for the enrichment analysis: minimum number of IDs in the category: 5; maximum number of IDs in the category: 2000; FDR Method: BH; significance level: Top 10.

Table S1. Overview of a head-to-head comparison of the results of the comprehensive characterisation of the two types of secretome.

**Secretome Composition**		
**Groups**	**Investigation (Method)**	**Results**
rbADSC Secretome	Total identified proteins (Proteomics, LC-MS/MS)	2774
rbADSC-rbTenocyte Secretome		2693
rbADSC Secretome	Differently expressed proteins (Proteomics, LC-MS/MS)	61
rbADSC-rbTenocyte Secretome		21
rbADSC Secretome	Upregulated proteins (Proteomics, LC-MS/MS)	Ras-associated binding protein 15 (RAB15)<br>Choline kinase β (CK β)<br>Fibronectin type III domain containing 3B (FNDC3B)<br>Amino oxidase (AMO)
rbADSC-rbTenocyte Secretome		Aggrecan (ACAN)<br>Biglycan (BGN)<br>Tenascin-C (TNC)<br>Collagen XIA1 (COL XIA1)<br>MMP-9<br>Angiopoietin-1 (ANGPT1)
rbADSC		Collagen I (COL I)

Secretome	Equally expressed proteins (Proteomics, LC-MS/MS)	Collagen III (COL III)<br>TIMP metallopeptidase inhibitor 1 (TIMP1)<br>Growth factors relevant for tendon healing<br>Cytokine transforming growth factor beta 1 (TGF- β)<br>Interleukin 6 (IL-6)
rbADSC-rbTenocyte Secretome		
rbADSC Secretome	Ligand-receptor pairs (Proteomics, LC-MS/MS)	none
rbADSC-rbTenocyte Secretome		Nrp2 → Pgf<br>Nrp2 → Sema3c<br>Nrp2 → Vegfa → Axl → Adam10 → Cadm1<br>Tek → Angpt1<br>Tek → Angpt2<br>Pdgfrb → Pdgfd<br>Tnc → Sdc4 → Adam12<br>Vldlr → Thbs1<br>Vldlr → Serpine1 → Plaur → Srpx2<br>Vldlr → App → Sorcs1<br>Kremen1→ Dkk3
rbADSC Secretome	Enriched molecular functions / biological processes (Proteomics, LC-MS/MS)	Transferase activity<br>Transmembrane transporter activity
rbADSC-rbTenocyte Secretome		Glycosaminoglycan (GAG) binding<br>Metal and cation ion binding
rbADSC Secretome	Over representation analysis (Proteomics, LC-MS/MS)	Nucleic acid and nucleotide binding
rbADSC-rbTenocyte Secretome		Ion binding, protein binding and structural molecule activity

Functional Characterisation – Secretome Efficacy *in vitro* and *in ovo*

Groups	Investigation (Method)	Results*							
		**6 h**	**1 d**	**2 d**	**3 d**	**4 d**	**5 d**	**7 d**	**10 d**
rbADSC Secretome	Metabolic activity (Alamar blue assay)				1.90		3.38	2.81	1.94
rbADSC-rbTenocyte Secretome					1.57		3.00	2.51	1.79
rbADSC Secretome	Scratch width, healthy environment (Migration assay)	0.90	0.65						
rbADSC-rbTenocyte Secretome		0.83	0.41						
rbADSC Secretome	Scratch area, healthy environment (Migration assay)	0.81	1.21						
rbADSC-rbTenocyte Secretome		0.61	1.06						
rbADSC Secretome	Scratch width, inflammatory environment (Migration assay)	1.08	1.33						
rbADSC-rbTenocyte Secretome		0.93	0.79						
rbADSC Secretome	Scratch area, inflammatory environment (Migration assay)	1.35	1.30						
rbADSC-rbTenocyte Secretome		1.00	0.90						
rbADSC Secretome	ALOX15 (Gene expression, qPCR)				0.72		1.56	0.85	
rbADSC-rbTenocyte					0.70		2.11	0.77	

Secretome						
rbADSC Secretome	BGN (Gene expression, qPCR)		1.35		1.08	0.75
rbADSC-rbTenocyte Secretome			1.24		1.26	0.62
rbADSC Secretome	COL I (Gene expression, qPCR)		0.62		0.43	0.65
rbADSC-rbTenocyte Secretome			0.52		0.71	0.72
rbADSC Secretome	COL III (Gene expression, qPCR)		0.69		0.46	0.53
rbADSC-rbTenocyte Secretome			0.78		0.80	0.47
rbADSC Secretome	IL-6 (Gene expression, qPCR)		69.64		223.29	286.92
rbADSC-rbTenocyte Secretome			76.51		91.51	109.10
rbADSC Secretome	Ki67 (Gene expression, qPCR)		0.82		2.44	1.11
rbADSC-rbTenocyte Secretome			0.91		3.57	1.26
rbADSC Secretome	MKX (Gene expression, qPCR)		0.50		0.84	0.59
rbADSC-rbTenocyte Secretome			0.52		1.22	0.37
rbADSC Secretome	MMP-2 (Gene expression, qPCR)		0.77		0.16	0.69
rbADSC-rbTenocyte Secretome			0.59		0.42	0.39
rbADSC Secretome	MMP-9 (Gene expression, qPCR)		3.72		4.76	13.41
rbADSC-rbTenocyte Secretome			1.59		4.30	3.78
rbADSC Secretome	aSMA (Gene expression, qPCR)		0.18		0.27	0.13
rbADSC-rbTenocyte Secretome			0.32		1.28	0.42
rbADSC Secretome	TIMP1 (Gene expression, qPCR)		2.46		1.89	1.09
rbADSC-rbTenocyte Secretome			3.00		4.41	1.16
rbADSCs Secretome	TNC (Gene expression, qPCR)		1.22		0.43	1.18
rbADSCs:rbTenocytes Secretome			1.66		1.03	0.66
rbADSC Secretome	TNMD (Gene expression, qPCR)		0.46		0.25	0.40
rbADSC-rbTenocyte Secretome			0.48		0.37	0.48
rbADSC Secretome	Survival	1		0.94		0.82

rbADSC-rbTenocyte Secretome	(Angiogenesis, CAM assay)	1	0.88	0.73
rbADSC Secretome	Junctions (Angiogenesis, CAM assay)	1.15	1.38	1.73
rbADSC-rbTenocyte Secretome		1.50	1.84	2.13
rbADSC Secretome	Total vessel length (Angiogenesis, CAM assay)	1.12	1.22	1.25
rbADSC-rbTenocyte Secretome		1.14	1.22	1.31
rbADSC Secretome	Vessel density (Angiogenesis, CAM assay)	1.13	1.26	1.64
rbADSC-rbTenocyte Secretome		1.55	1.70	2.10
rbADSC Secretome	Vessel hierarchy (Angiogenesis, CAM assay)	1.84	2.59	1.72
rbADSC-rbTenocyte Secretome		1.67	1.78	2.49
rbADSC Secretome	CAM thickness (Angiogenesis, CAM assay)			1.31
rbADSC-rbTenocyte Secretome				1.31

*Fold change secretome group versus control group (medium alone)
